# A Review of the Effects and Production of Spore-Forming Probiotics for Poultry

**DOI:** 10.3390/ani11071941

**Published:** 2021-06-29

**Authors:** Igor V. Popov, Ammar Algburi, Evgeniya V. Prazdnova, Maria S. Mazanko, Vladimir Elisashvili, Anzhelica B. Bren, Vladimir A. Chistyakov, Elizaveta V. Tkacheva, Vladimir I. Trukhachev, Irina M. Donnik, Yuri A. Ivanov, Dmitry Rudoy, Alexey M. Ermakov, Richard M. Weeks, Michael L. Chikindas

**Affiliations:** 1 Agrobiotechnology Center, Don State Technical University, 344000 Rostov-on-Don, Russia; ipopov@donstu.ru (I.V.P.); prazdnova@sfedu.ru (E.V.P.); mary.bio@list.ru (M.S.M.); brenanzhelika@yandex.ru (A.B.B.); vladimirchi@yandex.ru (V.A.C.); etkacheva@donstu.ru (E.V.T.); dmitriyrudoi@gmail.com (D.R.); amermakov@yandex.ru (A.M.E.); 2 Department of Food Science, Rutgers State University, New Brunswick, NJ 08901, USA; ammar.algburi@gmail.com; 3 Department of Biotechnology, College of Science, University of Diyala, Baqubah 32001, Iraq; 4 Academy of Biology and Biotechnology, Southern Federal University, 344090 Rostov-on-Don, Russia; 5 Institute of Microbial Biotechnology, Agricultural University of Georgia, 0131 Tbilisi, Georgia; v.elisashvili@agruni.edu.ge; 6 Moscow Timiryazev Agricultural Academy, Russian State Agrarian University, 127550 Moscow, Russia; rector@rgau-msha.ru; 7 Russian Academy of Sciences, 119991 Moscow, Russia; imdonnik@presidium.ras.ru; 8 FGBNY Federal Research Engineering Center VIM, 109428 Moscow, Russia; vniimzh@mail.ru; 9 Health Promoting Naturals Laboratory, School of Environmental and Biological Sciences, Rutgers State University, New Brunswick, NJ 08901, USA; rmw143@sebs.rutgers.edu; 10 Department of General Hygiene, I.M. Sechenov First Moscow State Medical University, 119435 Moscow, Russia

**Keywords:** poultry, spore-forming probiotics, *Bacillus*, antibiotics, growth performance, solid-state fermentation

## Abstract

**Simple Summary:**

Spore-forming probiotics are widely used in the poultry industry for their beneficial impact on host health. The main feature that separates spore-forming probiotics from the more common lactic acid probiotics is their high resistance to external and internal factors, resulting in higher viability in the host and correspondingly, greater efficiency. Their most important effect is the ability to confront pathogens, which makes them a perfect substitute for antibiotics. In this review, we cover and discuss the interactions of spore-forming probiotic bacteria with poultry as the host, their health promotion effects and mechanisms of action, impact on poultry productivity parameters, and ways to manufacture the probiotic formulation. The key focus of this review is the lack of reproducibility in poultry research studies on the evaluation of probiotics’ effects, which should be solved by developing and publishing a set of standard protocols in the professional community for conducting probiotic trials in poultry.

**Abstract:**

One of the main problems in the poultry industry is the search for a viable replacement for antibiotic growth promoters. This issue requires a “one health” approach because the uncontrolled use of antibiotics in poultry can lead to the development of antimicrobial resistance, which is a concern not only in animals, but for humans as well. One of the promising ways to overcome this challenge is found in probiotics due to their wide range of features and mechanisms of action for health promotion. Moreover, spore-forming probiotics are suitable for use in the poultry industry because of their unique ability, encapsulation, granting them protection from the harshest conditions and resulting in improved availability for hosts’ organisms. This review summarizes the information on gastrointestinal tract microbiota of poultry and their interaction with commensal and probiotic spore-forming bacteria. One of the most important topics of this review is the absence of uniformity in spore-forming probiotic trials in poultry. In our opinion, this problem can be solved by the creation of standards and checklists for these kinds of trials such as those used for pre-clinical and clinical trials in human medicine. Last but not least, this review covers problems and challenges related to spore-forming probiotic manufacturing.

## 1. Introduction

Spore-forming probiotics are gaining popularity in the poultry industry as natural growth promoters [1,2]. The most prevalent probiotics are lactic acid bacteria (LAB), lactobacilli, and *Bifidobacterium* spp., which are normally found in the gastrointestinal tracts (GIT) of animals and humans [3]. On the contrary, spore-forming bacteria, *Bacillus* spp. and *Clostridium* spp., due to their ability of encapsulation, can be found not only in GIT, but also in soil, water, and dust [4]. This makes the development process for spore-forming probiotics more accessible compared to LAB. Spore formation increases the survival of probiotics during the manufacturing process including fermentation, freezing, drying, thawing, and rehydration. Additionally, spores of these probiotics have a greater ability to survive passage through the gut and to proliferate and colonize the digestive tract [5,6]. This ability makes spore-forming probiotics an ideal feed additive for livestock, especially in the poultry industry.

There is an urgent need for an effective replacement for now-banned antibiotic growth promoters (AGPs). Alternatives currently under development are antibodies, prebiotics, bacteriophages, vaccines, and antimicrobial peptides [7]. However, we believe probiotics, especially spore-forming ones, are a suitable solution. Over the past several decades, they have demonstrated significant success not only in the control of pathogens, including drug-resistant strains [8,9], but also in natural growth promotion, improvement of feed conversion rates, and other zootechnical characteristics in broilers, laying hens, and other poultry species [10].

The main aim of this review is to summarize and discuss (a) the current achievements of microbiota studies in livestock birds, (b) the poultry health-promotion effects of spore-forming probiotics such as immune-modulation, (c) metabolism improvement, (d) interaction with hosts’ gene expression, and (e) the impact of spore-forming probiotics on productivity rates and egg and sperm quality. Important biotechnological aspects of spore-forming probiotics’ manufacture such as cultivation and solid-state fermentation will also be discussed.

## 2. Where to Start? Brief Diving into the Chicken’s Gastrointestinal Tract and Its Commensal Microbiota

Before considering the effects of probiotic bacteria on the gut microbiota, we should take a closer look at the commensal microbiota of the chicken GIT. Birds have a higher rate of passage of food through the GIT and increased activity of digestive enzymes compared to other vertebrates [11,12]. In the text below, we will study each part of the intestine separately.

### 2.1. Oral Cavity and Goiter

The oral cavities of birds do not contain teeth, unlike those of mammals, and therefore, food does not linger there, immediately going down the esophagus into the goiter. However, saliva production, which moistens food, occurs in the oral cavity, providing a moist and favorable environment for the development of microorganisms in the goiter [12].

A goiter is an enlargement of the esophagus where food can be stored before it enters the stomach. If the bird is hungry, food can enter the stomach, bypassing the goiter; if there is enough food, it will linger in the goiter and enter the stomach in small portions [13]. In addition to saliva from the oral cavity, a mucous secretion containing mucin is produced in the goiter. This secretion creates ideal conditions for softening food and the development of the microbiota, including microbial fermentation of food [12,14].

In chickens, the goiter microbiota is mainly represented by bacteria ingested with food. Their numbers can reach 10^9^ CFU/g [15]. These are primarily *Lactobacillus* and *Clostridiaceae*, *Bifidobacterium*, *Enterobacteriaceae*, and *Enterococcus* species [16]. Han et al. (2016) confirmed these data, showing that the goiter was dominated by Firmicutes (60%) followed by Bacteroidetes (14%), Cyanobacteria (13%), and Proteobacteria (8%). Among Firmicutes, *Lactobacillus* (28% of all species), *Bacillus* (4%), and *Bacteroides* (4%) were most prevalent [17].

A review by Feye et al. (2020) also showed that *Lactobacillus*, *Bifidobacterium*, and *Enterobacter* were most often represented in the chicken goiter. However, in free-range birds, large amounts of *Bacillus* (up to 76%) can also be found in the goiter [18].

Food can stay in the goiter for up to 14 h; however, it most often does so for 1–3 h. Next, it enters the stomach, taking a part of the microbiota with it, while the rest remains on the goiter walls [12].

### 2.2. Glandular Stomach and Gizzard

The glandular stomach, or proventriculus processes chyme, using enzymes at acidic pH. The pH can be 2.3–4.8 [13]. The food does not linger here for very long; most often, in chickens, the time spent for food in the glandular stomach is 10–30 min [12].

In the gizzard, food is broken down by small stones or grit. It is also where the first part of the enzymatic digestion of food and the bulk of its mechanical grinding takes place [15]. The muscular intestine contents are then transported to the small intestine in small portions [12].

Due to the low pH, the number and diversity of microorganisms in the gizzard are lower than in the goiter and intestines. The number of bacteria does not exceed 10^8^ CFU/g [15]. According to “Sturkie’s avian physiology,” it is possible for the contents of the small intestine to return to the gizzard [12]. Furthermore, this means the regurgitation of the microbiota of the small intestine. In general, the gizzard microbiota is represented mainly by *Lactobacillus* as well as Clostridiaceae, *Enterococcus*, and coliforms [16]. A review by Feye et al. (2020) stated that the main bacteria in the gizzard were *Lactobacillus*, *Enterobacteriaceae*, and coliform bacteria [14].

### 2.3. The Small Intestine

In the small intestines of birds, the duodenum, ileum, and jejunum can be distinguished; however, there are no significant functional differences between them, nor pronounced boundaries such as sphincters [13]. The pH gradually increases from 5.8 to 6.4 in the intestines [13]. In addition to the secretions of the pancreas and liver, the small intestine wall produces a secretion containing enzymes and mucin. On average, chickens have 2 to 8 h of food in the small intestine [12]. The total amount of microbiotas here increase significantly compared to other regions of the GIT, up to 10^9^–10^11^ CFU/g [15].

Despite the absence of clear boundaries, the microbiota in the different sections of the intestines are distinct. Apparently, this is due to a change in the composition of the available nutrients due to the intestinal wall’s enzymatic digestion and absorption. Thus, for example, although representatives of Firmicutes (>60%) and Bacteroidetes (>10%) are the predominant species in the small intestine as a whole, the duodenum also contains a high amount of Proteobacteria (>20%). In comparison, in the rest of the sections, Proteobacteria account for less than 10% of the total microbiota. In the ileum, representatives of Actinobacteria are most widely represented [19]. In terms of individual genera, lactobacilli can be isolated, which make up more than 35% of the small intestine’s microbiota. *Enterococcus* occupies a dominant position in the ileum (up to 30%); the highest numbers of *Corynebacterium* are also found there [19].

A study by Mohd Shaufi et al. also demonstrated the predominance of Firmicutes in the ileum. According to this work, Firmicutes accounted for 85% of the microbiota, and Proteobacteria were second in number. On the other hand, the dominant orders were *Clostridiales*, bacilli (including lactobacilli), and Gammaproteobacteria, mainly Enterobacteriales [20]. According to another study, the number of lactobacilli in the ileum can be as high as 70%, with the remaining dominant genera being Clostridiaceae (11%), *Streptococcus* (6.5%), and *Enterococcus* (6.5%) [21].

### 2.4. Cecum

Birds have two blind guts, and in chickens, they are well developed. Food stays in the ceca for 12–20 h [15,22]. It is difficult to determine the exact time because the contents are thrown and ejected back through the same opening near the small intestine transition into the rectum. The cecum contents are constantly mixing, maintaining a stable composition of the microbiota even under the conditions of a fast digestion rate in chickens [15,22,23]. Unlike the small intestine, where the main functions are digestion and absorption of nutrients, the cecum’s primary function is enzymatic activity and detoxification of harmful substances [24]. As a result of fermentation processes in the cecum, chickens receive biologically active substances such as short-chain fatty acids [25].

There are also a high number of microorganisms in the cecum, up to 10^11^ CFU/g [15]. Due to the intestinal contents’ long-term presence, the microbiota of the cecum is the most diverse among all parts of the intestine [19,20,21]. It forms a cluster that is distinct from the microbiota of the small intestine and rectum. According to a study by Xiao et al., Bacteroidetes were predominant in the cecum (>50%), while Firmicutes constituted only about 40%. The number of Lactobacillus species decreased in the cecum, while Bacteroides increased to more than 40% [19]. In another study, the dominant group was Clostridiaceae (65%) followed by *Fusobacterium* (14%), *Lactobacillus* (8%), and *Bacteroides* (5%). However, this study also highlighted a difference in the cecum and jejunum microbiomes [21].

Another genome-wide study showed the predominance of *Clostridiaceae* (>50%) and a high level of Bacteroidetes (about 20%) [20]. A study of metagenomes uploaded to public databases showed that most often, Firmicutes (78%) and Bacteroidetes (11%) prevailed in the cecum, which was consistent with the data of Shaufi et al. and Lu et al. [20,21]. Among the secondary groups, the most significant number of representatives related to Proteobacteria and Actinobacteria. Among Firmicutes, the most common were *Ruminococcus*, *Clostridium*, and *Eubacterium*; among Bacteroidetes, up to 40% were *Bacteroides*. At the phylum level, Proteobacteria, Desulfohalobium, *Escherichia*/*Shigella*, and *Neisseria* were the most abundant [26].

A study by Glendinning et al. (2020) also investigated the cecum microbiome. According to this study, Firmicutes were the dominant group, and their abundance was as high as 95%. Among Firmicutes, the majority were Clostridia (88%) followed by Lactobacillales (5%). The remaining 5% of the microbiota was distributed among Actinobacteriota, Proteobacteria (all *Escherichia coli*), Verrucomicrobiota, Bacteroidota, Campylobacterota, Cyanobacteriota, and Desulfobacterota [27]. Thus, according to various studies, the microbiota of the cecum of chickens can vary significantly.

### 2.5. Colon

The colon is short, and its contents stay inside for approximately one hour before entering the cloaca [13]. In general, the composition of the large intestine’s microbiota is closer to that of the small intestine than that of the colon. It is also dominated by Firmicutes (>60%) and Bacteroidetes (>10%). The most widely represented genera are *Lactobacillus* and *Enterococcus* [19,28]. Due to reverse peristalsis, the cloaca contents can enter the rectum along with uric acid, negatively affecting the colon microbiota and influencing the data obtained from fecal samples [12].

### 2.6. Differences in the Microbiota of Chickens and Factors Affecting Them

From the data presented above, we can conclude a wide variability in the poultry microbiome. Stanley et al. (2013) conducted a study comparing the microbiomes of broiler chickens raised under the same conditions, repeated three times. To compare the results obtained, the authors used QIIME v1.3.0 open source software. Various clustering methods provided in the QIIME package showed that the resulting microbiomes formed three clusters. Non-phylogenetic beta diversity metrics grouped samples from the three trials into three fully separated groups; the Spearman metric completely separated samples from trial 3 into two separated but close groups of samples originating from trials 1 and 2. Unweighted and Weighted Unifrac also showed some, but not total, separation of the samples from each trial. The authors attributed this level of variability, even under carefully controlled conditions, to the high levels of hygiene during egg incubation that destroyed the shell microbiota. Thus, instead of the mother’s shell microbiota, chicks could get bacteria from entirely different sources: egg transport boxes, staff, etc. These factors were unstable and difficult to control, and because of this, the resulting microbiota differed significantly between the repeats [29].

Oakley et al. (2014) carried out a broad comparison of various metagenomes of the ceca of birds and a comparison of their functional activity due to the genes present in the microbiome. They concluded that despite a high variability of taxa, functional variability within the chicken cecal microbiome was much lower and, to a much lesser extent, differed between samples. This meant that while the composition of the microbiota in different chicken groups could vary widely, the gut microbiota equally performed its primary functions [15].

The composition of the microbiota is highly dependent on the age of the bird. After hatching, the intestines of chicks are rapidly colonized by bacteria; however, as birds age, these alterations decrease upon reaching adulthood [21,30,31].

Diet also affects the gut microbiota of birds. Factors such as pellet size, choice of food grains, and the microbiota of those foods can cause shifts in birds’ microbial communities [32,33,34,35]. Antibiotics can selectively affect the gut microbiota, which leads to changes in the ratio of different groups of gut microorganisms [36,37]. Housing conditions can also affect the microbiota. When litter is reused, the microorganisms contained in it can affect the broilers’ microbiomes [38,39]. Pin Viso et al. (2021) analyzed the available metadata from MG-RAST and the NCBI Sequence Read Archive using QIIME v1.9.1 software. According to their analysis, there was a correlation between such factors as age, diet, and geographic location. The authors discussed the so-called “local microbiota” characteristics of different countries [40]. Other important factors influencing the microbiota of chickens are probiotics, prebiotics, and their compositions [41,42,43].

## 3. Chicken Probiotics: Why Spore-Formers?

As mentioned above, in the different regions of the gastrointestinal tract, the microbiota differs in quantity, composition, and properties [15,16]. Moreover, these differences can be seen by comparing the luminal and mucosa-associated gut microbiome [31,44]. Thus, not every microorganism can survive in a specific part of the gastrointestinal tract. This should be considered when looking for new probiotics. The best option, in this case, is to search for potential probiotics among the commensal microorganisms inhabiting the area of interest to us in the intestine of the host organism,. This will ensure a potential probiotic’s ability to colonize the necessary part of the gastrointestinal tract.

Other scientific groups seem to have come to the same conclusion. Adhikari et al. (2017), after studying lactobacilli from different intestinal ecotopes, concluded that bacteria already living in the same place as the planned probiotic were more likely to be potential probiotics [45]. On the other hand, many of the spore-forming bacteria considered probiotics are not permanent representatives of the intestinal microbiota, but live in different habitats. For example, *Bacillus* species usually live in soil and can be transported to other surfaces, together with dust [46]. They are also found in small numbers in the intestines of humans and other animals [47,48]. For example, studies in humans showed that the presence of bacterial spores in feces lasted longer than the expected transit time of food in the studied organism [49]. Moreover, in the 1980s, data were obtained that determined that bacilli spores could germinate in the lumen of the rabbit intestine [50]. The same data have been confirmed in mice and chickens [51,52]. It was shown that six times more bacilli were excreted from the GIT of mice than were obtained orally [51]. Increases in cell count also have been reported in pig studies, but to a lesser extent [53].

It was shown in a study using an artificial intestine model that various probiotic *Bacillus* strains not only proliferated from spores in the intestine (up to 97% of the germination cell), but were also metabolically active under these conditions [54,55]. It has been noted that spore germination occurs at a high rate; for example, in the study by Latorre et al. (2014), 90% of spores germinated in the small intestine of chickens within one hour [56]. The germination and metabolic activity of the vegetative cells of bacilli in the intestines should not be surprising because it has long been known that many strains of bacilli are not strict aerobes [57]. More striking is the fact that spores can not only grow in the intestine, but also re-sporulate in the lower intestine [58,59].

It is assumed that spore adhesion to the intestinal walls contributes to the retention of spores in the intestines. This ability is promoted by the hydrophobicity of both exospores and the spores themselves [60,61]. Another possible explanation is the incorporation of bacillus cells into the biofilms of other bacteria on the intestinal surface, which may be facilitated by the general ability of *Bacillus* species to form a dense biofilm [62]. It was shown that probiotic bacilli could attach to fibronectin and mucin in the intestine due to proteins such as S-layer components, flagellin, and cell-bound protease, and such adhesion of spores was higher than that of vegetative cells [63,64].

Their ability to exist, germinate, and sporulate under the anaerobic conditions of the GIT allows us to speculate on bacilli as commensals of the intestines of humans and animals [62,65]. All of the above allows us to propose using bacilli isolated from animals’ intestines as spore-forming probiotics, prioritizing them over other probiotics if they are proven as safe for the mentioned application.

## 4. Spore-Forming Probiotics and Improving Poultry Health

In today’s poultry industry, microbial infections represent a major economic concern that requires an immediate response to prevent such infections around the world [66]. AGPs have been used since the 1940s as an innovative protocol to control pathogenic infection and ensure the healthy growth of broilers. However, AGPs have been banned in Europe since 2006 due to the emergence of microbial resistance to commonly used antibiotics. Recently, direct-fed microbials (DFMs) have been used instead of AGPs as alternative strategies in animal feed [67,68]. DFMs are “live microorganisms which, when administrated in adequate amounts, confer a health benefit on the host” [69]. Several studies have recommended the use of DFMs for improving broilers’ immune response and growth performance [70,71,72].

Feed enzymes such as protease and carbohydrase play a key role in reducing indigestible feed molecules such as indigestible proteins, which are a source of nutrients for pathogenic bacteria [73]. Indeed, a combination of DFMs and commercial poultry diet enzymes has been reported as an effective protocol for improving the growth and gut health of broilers [74,75]. A recent publication of Dersjant-Li et al. (2013) referred to the beneficial economic impacts of using DFMs containing spore-forming *Bacillus* spp. as an alternative to AGPs for commercial production and health promotion in broilers. Therefore, more attention should be given to allochthonous probiotic groups, especially spore-forming *Bacillus* spp. These microbes are characterized by their hydrophobicity, autoaggregation and mucin adhesion, and their ability for long-term storage as spores [76].

### 4.1. Immuno-Modulation by Spore-Forming Probiotics

The spores of *Bacillus* species can be found almost everywhere: in soil, water, and dust. They commonly inhabit the guts of humans and other animals and have been experimentally isolated from fecal sampling [77]. After ingestion of food contaminated with endosymbiotic spores, the spores germinate, adhere, survive, and proliferate within the GIT [78]. The growth and metabolic activity of spore-forming bacteria are driven by their vegetative state when adequate nutrition is available [79].

The probiotic potential of spore-forming bacteria has been reported in several studies that have shown their ability to stimulate and/or modulate the poultry immune system by producing cytokines and immune defense substances. *Bacillus* DFMs in poultry were reported to play a role in immune modulation, proinflammatory cytokine production, and macrophage activation without cytotoxicity. After consumption of foods harboring spores, the spores could invade the primary lymphoid tissues of Peyers Patches and mesenteric lymph nodes [80]. These spores germinated inside the phagocytic cells, while vegetative genes were expressed [81]. After phagocytosis, the titer of anti-spores IgG, in addition to IgA, in the poultry serum, was elevated along with type 1 (Th1) T-cell responses [81]. Several studies reported that serum IgA and IgG were elevated in poultry groups treated with DFM Bacilli, indicating modulation of the humoral immune response [81,82]. The presence of phagocytic cells is necessary for the initiation and mediation of innate and adaptive immunity. Phagocytes produce the proinflammatory cytokines IL-1β, IL- 6, IL-8, and TL1A (homolog of TNF-α) in addition to the T-helper cytokines IL-12 and IFNγ, in the GALT and secondary lymphoid organs [83].

Cytokines are low-molecular weight peptides that play a basic role in the regulation of innate and adaptive host immune responses. After broiler chickens are fed with dietary *B. subtilis*, specific cytokines are produced including IL1β, IL12, and IFNγ, which are highly up-regulated and related to enhancing the protective immune responses to coccidiosis [84]. IL1β is a proinflammatory cytokine mediating innate immunity and is produced by macrophages, monocytes, and dendritic cells. IL12 is an essential cytokine for the initiation, differentiation, and regulation of cellular immunity and resistance to many pathogens [85]. Platzer et al. (1995) and Xu et al. (2012) reported that *B. subtilis* stimulated IL-10 and IL-4 production. When the concentration of proinflammatory cytokines was elevated, a negative feedback loop was activated to control the acute inflammatory response [86,87].

In healthy chickens fed with DFMs, the *Bacillus* strain contributed to the up- or down-regulation of various cytokines. Compared with the control groups, the up- and down-regulation of the gene expression of NO as well as several proinflammatory cytokines including IL-1β, IL-6, IL-8, and IFNγ were reported in healthy chickens treated with cocktails of multiple strains of bacilli [70]. These data confirmed the importance of the correct selection of DFM strains for poultry production [88].

Dietary DFMs were used by Lee et al. (2010), who found that dietary *B. subtilis* improved innate and acquired immunity in correlation with a reduction in induced avian coccidiosis in broiler chickens [70,89]. Dietary *Bacillus*-based DFMs were found to modulate host immunity and reduce the clinical signs of enteric infection by *Salmonella* spp. or *Clostridium* spp. in poultry [90,91,92,93]. Recently, Rajput et al. (2013) and Lee et al. (2015) reported that feeding broiler chickens a *Bacillus*-based DFM significantly stimulated inflammatory and anti-inflammatory cytokines against microbial infections such as coccidiosis and *C. perfringens*-associated infections. The authors concluded that increased body weight in *B. subtilis*-fed chickens was accompanied by increased expression of most innate immunity genes involved in several microbial infections [94,95]. Lee et al. (2015) found that broiler chickens fed with *B. subtilis* DMFs at 14- and 28-days post-hatch gained significant weight and exhibited a significant reduction in *Eimeria*- and *C. perfringens* necrotic enteritis toxin antibodies. In addition, the expression levels of genes encoding IL1β, IL12, and IF-γ were higher compared to non-treated control groups [95].

Considerable changes in the transcriptional expression of mRNA isolated from the mid-intestine have been induced. Bio-functional analysis detected 37 genes associated with “inflammatory response”. These data indicated that *B. subtilis* DFM could augment and improve the innate and cellular immunity of broiler chickens. Genome-wide transcriptional changes were studied in broiler chickens fed with dietary *B. subtilis* to provide sufficient data for the analysis of biological function. Lee et al. (2015) noticed that genes associated with the inflammatory response in *B. subtilus*-fed poultry were altered in the classification of “Disease and Disorders”. Examples included inducible nitric oxide synthase (iNOS), which was rapidly induced and led to the production of nitric oxide (NO) when exposed to allergens, oxidants, or cytokines [95,96]. Similar results were noticed after treatment with *B. subtilis* and Bifidobacterium: NO production increased in chicken intra-epithelial lymphocytes in the presence of *E. coli*, and IL-1β and IFNγ were up-regulated in chickens with *Clostridium* spp. infections [70,97,98,99,100]. NO plays a major role in the immune response, autoimmune processes, and the control of infectious diseases [101]. Defense molecules such as NO are produced as a result of the secretion of proinflammatory cytokines. low concentrations of NO and iNOS stimulate the maturation and production of immune cells. At the same time, high concentrations of NO have a destructive effect on DNA, lipids, and the protein composition of bacterial pathogens [102].

Moreover, an up-regulation of tumor necrosis factor (ligand) super-family member 15 (TNFSF15) was identified in *B. subtilis* DFM chickens and was validated by qRT-PCR [70]. TNFSF15 is a local proinflammatory cytokine in chickens involved in (i) stimulating T cell proliferation and (ii) inducing the production of IFNγ and granulocyte–macrophage colony-stimulating factor [83,85]. In addition, it was noticed that IFNγ production was improved when TNFSF15 was combined with IL-12/IL-18 in peripheral blood T cells and NK cells [103]. Further studies are required to investigate the relationship between DFMs, the immune response, and growth traits, especially in regard to some studies showing the prolonged effects of IL-1, TL1A, and IL-6. The effects were reported to cause a reduction in muscle cell translational efficiency due to inhibition of myogenic differentiation, which led to muscle proteolysis and a reduction in muscle mass [104].

### 4.2. Improvement of Metabolic Activities by Spore-Forming Probiotics

Regarding the beneficial effects of probiotics on intestinal health, spore-forming probiotics can play a role in (i) removing free-radicals [105], (ii) up–down regulation of mucin gene expression [106], and (iii) production of bacteriocins [107] and other antimicrobial substances that inhibit the growth of enteric pathogens and/or their virulence factors [108,109]. Flint and Garner (2009) reported that *B. licheniformis* and *L. bulgaricus* improved the digestibility of amino acids, protein, and starch [110].

There is a general agreement regarding the modulation of the composition of the normal gut flora by probiotics, which can improve feed conversion rates and eventually enhance digestion and absorption of nutrients by catabolizing substrates [111].

Mucin2 (MUC2) is a major mucin secreted in the gastrointestinal epithelial tissue of poultry in order to maintain a suitable thickness of the intestine mucosal layer, which is frequently sloughed off by intestinal movement and the actions of chemical and microbially derived substances [112,113]. Mucin is an important source of carbohydrates and exogenous nutrients for the growth and maintenance of intestinal flora [114]. The composition, secretion, and dynamics of intestinal mucin are affected by microbial colonization. The microbial community in the GIT could play a key role in mucin biosynthesis and/or degradation [115].

Aliakbarpour et al. (2012) found that birds fed a diet containing a probiotic strain of *Bacillus subtilis* significantly increased gene expression of intestinal MUC 2 mRNA in comparison to the control group. The higher the expression of the MUC2 gene, the more growth performance and improved intestinal morphology were reported in the *B. subtilis* probiotic-fed chicks. After the birds were fed a diet containing probiotics, the authors reported an increase in mucin gene synthesis, which positively influenced microbial interactions and numbers of mucosal cells in the GIT and eventually elevated efficiency of nutritional absorption [106].

The presence of ammonia in fecal material contributes to manure malodor, which is a major environmental problem associated with the poultry industry [116]. Solutions to such challenging issues are urgently required because of their adverse effects on the health status of animals and workers [117]. The biological activity of the intestinal microbiota and the chemical composition of nutrients are related to the malodor of ammonia emissions from feces. Dietary *B. subtilis* supplementation has been reported to enhance the enzymatic activity of the intestinal microflora, increasing their nitrogen utilization and eventually reducing ammonia emission in poultry feces [118]. Jeong and Kim (2014) noticed a significant reduction in ammonia emission without affecting nitrogen digestibility when *Bacillus* strains BS300 and BS600 were used. In addition, the authors found that food supplementation with *B. subtilis* did not influence the numbers of white blood cells, red blood cells, or lymphocytes. However, the exact mechanism of reducing ammonia production in feces has not been fully elucidated yet [119].

Several studies have referred to the capability of some *Bacillus* species to produce beneficial enzymes such as proteases, lipases, cellulases, xylanases, phytases, and amino acids [56,120,121]. The “anti-nutritional” factors within feed ingredients could potentially be neutralized by using such enzymes. Moreover, these enzymes play a key role in the absorption of nutrients through reducing intestinal viscosity by catalyzing indigestible starch polysaccharides. *Bacillus subtilis* producing subtilosin, catalase, and lactic acid was proven to enhance the growth of beneficial microorganisms including *Lactobacillus* species [122,123,124].

Several spore-forming bacilli have been isolated and identified as probiotics, but only a few strains are used commercially including *B. subtilis*, *B. licheniformis,* and *B. cereus* [125]. Recently, *B. amyloliquefaciens* B-1895 was reported to have positive health effects on poultry, increasing meat mass as well as food digestion and absorption in broilers [126,127]. Farhat-Khemakhem et al. (2018) determined the carbohydrate fermentation profile and enzymatic activities of *B. amyloliquefaciens* US573, using API-ZYM and API 50CHB kits. The author referred to the synergistic activity of a combination of enzymes that act in concert to degrade non-starch polysaccharides and phytates within feed components. These enzymes were also able to neutralize anti-nutritional factors and facilitate the absorption of nutrients [128].

Within the GIT, bacilli strains have been shown to participate in the metabolism of dietary substances and maintain intestinal homeostasis through the production of xenobiotics and antimicrobial compounds [129,130]. Endo et al. studied the effects of a mixture of probiotics including *Bacillus* and *Clostridium* spp. on the metabolism of lipids, the commensal cecal microflora, and other metabolites in cocks. The authors noticed a reduction in cholesterol levels in the liver and serum of the cocks when they were fed with a cholesterol-enriched diet containing a mixture of probiotics. In comparison to the control group, the chemical properties of the cecal material were altered and influenced by the different probiotics spp. that were incorporated into the diet [131].

Despite the above-mentioned beneficial effects of using spore-forming probiotics, we have not found answers to any of our raised questions in recent publications, specifically (i) efficient strategies for probiotic delivery to the intestinal tract, (ii) the physiological nature of probiotic interactions with the intestinal tissue, (iii) the timeline of probiotic bioavailability in the GIT (from germination until production of their beneficial effect), and (iv) the possible adverse effects of probiotic administration on the intestinal physiology of the tested birds.

### 4.3. Spore Formers in Health Promotion

Spore formation and antimicrobial production by some probiotics extend their survival and improve health benefits in different habitats [132]. In addition to bacteriocins, the genus *Bacillus* can produce several antimicrobial substances such as peptides, lipopeptide antibiotics, and non-modified bacteriocins [133]. Spore-forming probiotics have been used for several years in poultry and aquaculture feeding to prevent oral and gastrointestinal infections [134]. *Bacillus*–produced antimicrobials have broad-spectrum potential against human and animal pathogens including yeasts, fungi, and Gram-negative and Gram-positive bacterial species.

## 5. Control of Microbial Pathogens

The ability to control pathogenic bacteria is a major concern in the animal industry, especially in poultry production. In the United States, *Salmonella*, a pathogen of the GIT in poultry, causes approximately 1 million illnesses every year. Furthermore, *Clostridium* spp., a zoonotic pathogen, costs the USA about $6 billion annually [135]. Several factors affect the bio–physiological activity of pathogens in the gastrointestinal tract. The mechanisms behind the reduction in pathogenic activity due to the actions of the normal commensal microbiota is not yet understood. However, as mentioned before, *Bacillus*-based DFMs could directly inhibit pathogenic growth in vitro by competitive inhibition, production of AMPs, or enhancement of the intestinal mucosa to prevent microbial dissemination across the membrane [136]. The *Bacillus* genera was identified more than 50 years ago, and *Bacillus* spp. utilize up to 5% of their genome to produce several AMPs, which have been purified and commercialized globally [137,138,139]. These AMPs may have either a narrow or broad range of antimicrobial activity against closely related organisms [138]. The killing mechanism of AMPs is related to disruption of the bacterial cell membrane, an efficient strategy for inhibiting or preventing bacterial growth [140].

Interestingly, the spores of *Bacillus subtilis* have been used as probiotics for both human and animal consumption [141]. In the agricultural industry, bacterial spores are being used as treatments and potential alternatives to antibiotics for oral infections and intestinal disorders, but only under clinical supervision. There is a general agreement around the capability of the gut microflora to impair the colonization of the GIT by pathogenic bacteria. Basically, there are three strategies to prevent pathogen–intestinal colonization: (i) elimination of pathogenic bacteria by the immune system, (ii) synthesis and production of antimicrobial substances, and (iii) competitive adhesion [142].

A study by Teo and Tan (2007) reported the antagonistic capability of *B. subtilis* strains after 24 h of incubation against *C. perfringens* ATCC 13124, the bacterial cause of necrotic enteritis (NE) in chickens [143]. Knap et al. (2010) found a reduction in both clinical and sub-clinical signs and mortality in NE-infected chicken treated with *B. licheniformis*. Similar effects were noticed when birds were treated with virginiamycin (50 g/ton) [144]. In the same regard, Craven (2000) reported that *C. perfringens* colonization was inhibited when broiler chickens were treated with probiotics [145]. In addition, Kaldhusdal and Lovland (2000) noticed a delay in *C. perfringens* colonization and the appearance of NE lesions in post-hatch broilers when they were directly fed with microflora that were isolated from adult birds [146]. In a field study, Park and Kim (2014) reported a significant decrease in the CFU/g of *S. typhimurium* in the presence of *B. subtilis* B2A in the chicken GIT [147]. Kadaikunnan et al. (2015) suggested *B. amyloliquefaciens* as a promising candidate for safe pharmaceutical applications. The spore-forming bacilli *B. amyloliquefaciens* was significantly active against all tested bacteria including *Bacillus subtilis*, *Enterococcus cloacae*, *Staphylococcus aureus*, and *S. epidermidis* [148]. Spores of *B. subtilis* showed a suppressive potential on the growth of *Escherichia coli* 078:K80 in a 1-day-old-chick model [149]. Upadhaya et al. (2016) found that the numbers of *Salmonella* and *E. coli* in the excreta were significantly lower (*p* < 0.05) in laying birds fed with bacilli strains (T1 and T2) compared to the control group. The author also noticed an increase in the counts of lactobacilli spp. in the small and large intestine when laying chickens were fed with *Bacillus subtilis* and *Bacillus methylotrophicus* [150]. In the same study, it was suggested that the reduction of pathogenic bacteria was related to antimicrobial production in addition to the enzymatic activity of lactobacilli spp. Barbosa et al. (2005) isolated several species of bacilli from broiler feces during their field study including *B. licheniformis*, *B. pumilus,* and *B. subtilis,* which were active against *C. perfringens* in vitro [123]. In the same regard, the antimicrobial activity of a *Bacillus cereus* strain isolated from soil against *C. perfringens* was reported by Bizani and Brandelli (2002). The activity of the bacilli strain was linked to the antibacterial effects of the bacteriocins produced during the exponential phase of growth [151].

*Bacillus* species are reported as biological sources of bioactive molecules including bacteriocins, therapeutic AMPs, and enzyme inhibitors, which could be used in pharmaceutical applications. These substances are effective antimicrobials against Gram-positive bacteria, Gram-negative bacteria, and filamentous fungi. *B. thuringiensis* produces thuricin, which actively inhibits *C. difficile* growth [152]. Teo and Tan (2005) identified a highly heat-stable proteinaceous substance produced by *B.subtilis* strain PB6 having potential antimicrobial activity against *C. perfringens* [153].

Bacteriocins, which are small, ribosomally synthesized molecules, are classified according to their structure, size, and post-translational modifications [107]. They have highly specific antimicrobial activity toward pathogenic bacteria without inhibiting the commensal gut microflora. Several bacteriocin-producing probiotics have been reported to have beneficial effects in broilers. For example, *Pediococcus pentosaceus*, *Lactococcus lactis*, and *Ruminococcus gnavus* have been shown to produce pediocin A, nisin, and ruminococcins A and C, respectively. These bacteriocins have been purified, and their antimicrobial activity against *C. perfringens* has been evaluated in vitro [154,155,156,157]. Bacteriocin-producing probiotics had an inhibitory effect on *C. perfringens* spores. Furthermore, *Brevibacillus borstelensis* was identified to have anti-C. perfringens activity, which could be ascribed to a thermostable, bacteriocin-like inhibitory substance (BLIS) [158].

In addition to controlling pathogenic bacteria, a study by Kadaikunnan et al. (2015) revealed that spore-forming *B. amyloliquefaciens* were significantly active against all tested fungi (*Aspergillus clavatus*, *A. fumigates*, *A. niger,* and *Gibberella moniliformis*) [148]. Spore-forming bacilli probiotics were reported to reduce the effects of *Eimeria* spp, the fungal agent of coccidiosis. It is estimated that worldwide annual loss due to this parasite is $3 billion [159,160]. Lee et al. (2010) showed a significant reduction in intestinal lesions caused by coccidiosis when three strains of *B. subtilis* were used as a DFM in broiler chickens [89]. In addition to reducing the clinical signs of the parasitic infection, Gadde et al. (2017) noticed a reduction in post-mortem observations of coccidiosis and enhancement of the immunological response toward the infection in the treated group that received *B. subtilis* [161]. It was recognized that *Eimeria* spp. promoted and magnified necrotic enteritis caused by *C. perfringens* [71].

*Bacillus* spp. are able to produce a series of AMPs from more than 300 different precursors through a series of peptide synthases. Bacitracin and gramicidin, which are non-ribosomal AMPs, are the most well-studied and popular antimicrobials produced by *Bacillus* species [162]. Furthermore, *Bacillus* spp. produce iturin and fengycin lipopeptides, which have demonstrated effective antifungal activity [163]. Several reports have been published on the antifungal activity of spore-forming probiotic metabolites against *C. albicans*, *Cryptococcus neoformans*, *T. mentagrophytes*, and *A. fumigates* [164]. *B. amyloliquefaciens* strains were found to produce surfactin, iturin, and fengycin [165]. The antifungal activity of these peptides was ascribed to alterations in cell membrane permeability [166]. Lavermicocca et al. (2003) found that 3-phenyllactic acid produced by Bacillus spp. showed an inhibitory effect on the growth of *A. ochraceus*, *P. roqueforti*, and *P. citrinu* [167]. In the same regard, Sjogren et al. (2003) reported that 3-hydroxy fatty acids produced by the tested bacilli showed significant antifungal activity against pathogenic molds and yeasts [168].

## 6. Spore-Forming Probiotics: Benefits for the Poultry Industry

As for the specific effects of spore-forming probiotics in poultry, preparations based on *B. licheniformis* have been widely used in the poultry industry for more than three decades, positively affecting feed conversion rates [169]. The range of drugs that can achieve this effect is still expanding. It has been shown that the use of preparations based on *Bacillus subtilis* equally improves the growth and productivity of broilers, and the effects are comparable to the results of the use of the antibiotics bacitracin and avilamycin. Among other things, probiotic preparation has been shown to positively affect the histomorphometry of the intestinal villi [170]. Most often, probiotics have a positive effect on the parameters of weight gain and the efficiency of food consumption in broilers [171,172,173]. According to the literature, spore-forming probiotics affect the following parameters measured in the poultry industry:

- Biochemical blood parameters showing the intensity of carbohydrate and protein metabolism (protein, glucose, urea) [174];
- Hematological blood composition (number of blood cells) as well as stimulation of the hematopoietic organs [175,176];
- Dynamics of live weight (weight gain) [177];
- Feed conversion rate (this appears to be increased by improved digestion and absorption of nutrients, leading to increased productivity) [178];
- Quantitative and qualitative composition of the microbiota [179];
- The level of oxidative stress (mRNA expression of antioxidant genes, oxidative damage index, etc.) [180];
- Meat quality (pH, cooking loss, shear, color, short-chain fatty acids, taste) [181];
- Egg production [182].
- Egg quality (yolk cholesterol, improved shell thickness, egg weight) [183];
- Sperm quality (volume of ejaculate, total number and concentration of spermatozoa in the ejaculate, number of morphologically abnormal cells in the ejaculate) [184];
- Intestinal barrier function [185,186].

All of the above-mentioned effects of spore-forming probiotics on poultry health and performance parameters are linked, as a specific and selective probiotic has not yet been identified that is associated with only one effect. The systematic action of probiotics is mediated by modulation of the GIT microbiota, resulting in a wide range of improvements to poultry performance.

It should be mentioned that there are currently no standards for probiotic poultry trials, specifically regarding definitions related to production performance parameters. Certainly, most of the in vivo probiotic research in poultry has followed the principles of blind randomization and placebo control, and has chosen suitable statistical tests for analysis. The most proven guideline for animal studies is ARRIVE (Animal Research: Reporting of In Vivo Experiments) [187]. However, it covers general items regarding study design and reporting results. In our mind, specific guidelines for probiotic poultry studies with the enumeration of essential parameters that should be evaluated in this kind of research must be created. They should include a set of minimum and possibly inexpensive parameters that must be studied to grant research reproducibility and comparability of results. As of now, there are a wide range of studies that cannot be compared because the authors did not report some production performance or quality characteristics in animals treated with spore-forming probiotics. For example, Ermakova et al. (2021) described the effects of *B. subtilis* probiotic on the yolk quality of Pharaon quail without mentioning other egg quality parameters such as egg weight, egg shape index, eggshell color, eggshell strength, yolk weight, eggshell weight, and other factors which did not require expensive equipment or consumables to evaluate, which makes it impossible to fully compare the results of this study with others [188]. Deng et al. (2012) did not report feed conversion rates, but they did provide egg weight and feed intake rate. As for the study design, the authors only provided information about heat stress-challenged birds treated with probiotics without any data about a positive control group with probiotic treatment and without experimental conditions [189]. Many examples of studied performance parameters including egg and sperm quality characteristics are provided in Table A1, Table A2 and Table A3. Moreover, it should be mentioned that most of the studies have not provided detailed information about spore-forming probiotics preparation, which undoubtedly affects the reproducibility of the studies.

## 7. Spore-Forming Probiotics Manufacturing, Exploiting Their Biosynthetic Potential

### 7.1. Cultivation Conditions for Bacillus spp. Growth and Spore Production

The global probiotics market surpassed USD 44.2 billion in 2019 and is expected to grow at a 7.7% compound annual growth rate to hit USD 74.3 billion by 2025 [190]. The use of *Bacillus* species as probiotic formulations is also rapidly expanding, requiring them to be produced in large quantities at a low cost. A key step in the development of a bioprocess is the production of bacteria with high yield and sporulation efficiency. Therefore, to obtain new and deeper fundamental knowledge about the physiology of bacilli and the sporulation process as well as to develop industrially significant technologies for the production of probiotics, various approaches and strategies have been used including the search for new spore-forming bacteria, the use of cost-effective plant materials as growth substrates, the optimization of fermentation media and cultivation conditions, and the development of improved bioprocess technologies [191,192,193,194,195,196,197,198,199]. Nevertheless, current knowledge on the physiology of probiotic *Bacillus* spp. production is still too limited to effectively realize their biotechnological potential on an industrial scale.

In laboratory studies, chemically defined synthetic media are frequently used for growth and sporulation. Although such media provide well-reproducible and homogeneous spore preparations, they are relatively expensive and provide a relatively low spore yield (1 × 10^8^–1 × 10^10^ CFU/mL). The concentration of the carbon source can play a decisive role in the process of sporogenesis by individual bacilli because it has been shown that with an increase in glucose concentration, the concentration of vegetative cells increases, but initial glucose concentrations above 5 g/L inhibit sporulation and sporulation efficiency decreases [191,193,200,201,202,203,204,205,206,207,208,209,210,211]. It has been suggested that depletion of the carbon source is the main stimulus for sporulation by *Bacillus* spp.; if that is the case, the concentration of the carbon source in the culture medium needs to be reduced to increase the sporulation efficiency and spore yield [5].

Although there is very little information in the literature on the effect of lignocellulose substrates on the formation of *Bacillus* spores, a significant number of publications are devoted to the study of solid-phase fermentation processes of spore-forming bacteria in the context of developing solid-phase processes related to the disposal of organic waste including various lignocellulose substrates such as straw, leaf-stem mass, pulp, and meal [197,198,199,201,202,203,204,205,206,207].

The use of solid-state fermentation (SSF) is an important tool in the prevailing circular bioeconomy paradigm, wherein organic solid waste is converted into value-added products. We believe that products obtained from waste by biotechnological methods differ favorably from traditional chemical sources in that the raw materials for their production are renewable raw materials of animal and plant origin, and the use of agro–industrial waste and by-products as substrates for the growth of probiotic microbes is one of the best ways to reduce production costs.

It is appropriate here to mention another area closely related to the prospects of waste recycling. SSF for plant disease biocontrol is considered to be one of the most promising alternatives to chemicals and is being commercially developed in many countries where *Bacillus* strains have a significant background [196,208,209].

However, it is necessary to identify the species and even the strain-specific lignocellulosic material to maximize the probiotic potential of spore-forming *Bacillus* spp. Thus, cornmeal and soybean meal positively influenced spore production by *B. amyloliquefaciens* BS-20, while no significant effects were found from wheat bran and molasses [195]. An optimized medium containing glucose, corn meal, soybean meal, and beef extract provided an 8.8-fold increase in spore yield compared with a control medium. In another study, a combination of tapioca with lactose in a nutrient medium for submerged cultivation of *B. amyloliquefaciens* B128 resulted in a spore yield of 5.92 × 10^8^ spores/mL [212]. A wide range of lignocellulosic materials with different chemical compositions have been used to evaluate *B. subtilis* KATMIRA1933 spore production under submerged fermentation conditions [201]. Milled soybean and sunflower processing by-products resulted in good growth of bacilli and accumulation of vegetative cells but failed to promote mass sporulation as compared to a control medium. On the contrary, mandarin peels followed by ethanol production residue (EPR) from corn grains provided an especially high yield of spores (5.7 × 10^10^ and 2.9 × 10^10^ spores/mL, respectively). Interestingly, the number of spores (4 × 10^9^ spores/mL) increased by 7 and 10 times with an increase in the concentration of mandarin peel in the medium from 10 to 30 and 40 g/L, respectively. Further increases in mandarin peel concentration did not favor spore formation. Moreover, the authors found that using cheese and curd whey instead of distilled water to prepare a culture medium containing mandarin peel, EPR, or a mixture thereof accelerated the initial growth of the bacilli and increased the spore yield to 5.8–7.4 × 10^10^ spores/mL. Likewise, *B. amyloliquefaciens* B-1895 appeared to be an efficient spore-forming bacterium producing 8.2–10.8 × 10^9^ spores/mL in the submerged fermentation of corn cobs, EPR from wheat grain, wheat bran, sunflower extraction cake, and mandarin peels [201]. It can be inferred then that these substrates contain all the nutrients required for both bacterial growth and effective sporulation. In addition, during the fermentation of these materials, *B. subtilis* KATMIRA1933 and *B. amyloliquefaciens* B-1895 exhibited relatively low endoglucanase and xylanase activities, which hydrolyze lignocellulose polysaccharides to metabolizable sugars to provide bacterial cultures with their necessary carbon sources. Consequently, only traces of reducing sugars were detected, even at the end of submerged fermentation, when the bacterial metabolism and proliferation had significantly declined. These circumstances may lead to the prevention of sporulation inhibition caused by elevated concentrations of sugars. On the whole, comparative analysis of the data received showed that in the submerged fermentation of lignocellulosic materials, both bacilli produced higher yields of spores as compared with those in the glucose-containing medium. Thus, these results indicate that various lignocellulosic materials may be successfully exploited as growth substrates for the cultivation of spore-forming bacteria.

Typically, plant materials, being a rich carbon source, contain nitrogen in concentrations that are suboptimal for the cultivation of microorganisms, which makes it necessary to include an additional nitrogen source for their optimal growth. Several studies have shown that both the nature and concentration of nitrogen sources are crucial nutritional factors affecting bacilli growth and spore production in both synthetic and lignocellulose-based media [200,212,213,214]. In particular, mandarin peels represented an excellent growth substrate for *B. subtilis* KATMIRA1933 growth and spore production, providing an accumulation of 2 × 10^10^ spores/mL [13]. However, supplementation of this medium with peptone ensured a three-fold increase in the spore yield, whereas ammonium sulfate sharply inhibited the sporulation process. Moreover, the number of produced spores changed to 8.3 × 10^10^ spores/mL when the nitrogen concentration in the nutrient medium was increased to 40 mM. The authors attributed the positive effect of peptone to the higher production of bacterial biomass and increased sporulation efficiency. In the cultivation of *B. amyloliquefaciens* B-1895, corn cobs appeared to be an excellent growth substrate, providing an accumulation of 7.2 × 10^9^ spores/mL [201]. Supplementation of the medium with casein hydrolysate at a concentration of 20 mM as the nitrogen led to a three-fold increase of spore numbers. Chen et al. (2010) achieved a maximal spore yield of 1.56 × 10^10^ spores/mL after 40 h cultivation of *B. subtilis* WHKZ12 in a 30 L fermenter using cornstarch, wheat bran, corn flour, corn steep liquor, soybean flour, and yeast extract at optimal concentrations. Overall, the data received indicate that the determination of an optimal nitrogen source and concentration are necessary for the best growth and sporulation of *Bacillus* species, and that a consideration of the individual physiological parameters of each strain must be taken into account [201].

### 7.2. Fermentation Methods for the Production of Probiotics

#### 7.2.1. Solid-State Fermentation

Probiotic yields and the cost of their production depend significantly upon the method of plant raw material fermentation. According to several studies, the preparation of probiotics using solid-state fermentation (SSF) is both cost-effective and environment-friendly [6,215,216,217,218].

The exploitation of biofilm growth is the key feature determining SSF’s advantages because biofilms are perhaps the most natural form of microbial communities’ existence [219,220]. Bacteria assembled in a consortium are considerably persistent, just as an organized community is much stronger than a group of separated individuals in higher-level organisms. As is known, the high persistence of pathogenic biofilms creates a number of problems in the treatment of infections. However, this phenomenon has a second side. Probiotic bacteria combined in biofilms grow better, are more resistant to drying, and can colonize the gastrointestinal tract of the host more efficiently [221].

SSF of plant raw materials is attractive compared to the submerged fermentation process because its implementation requires relatively low investment and less sophisticated equipment. It is easy to handle and has higher productivity and concentration of the final product, which can be dried directly without centrifugation, as well as a low wastewater output. Therefore, the cost-effectiveness of SSF is not as dramatically dependent on scaling as in the case of liquid-state fermentation. Moreover, we believe that the indisputable advantage of solid-phase fermentation is the possibility of its organization precisely within the framework of a small-scale technology focused on local raw materials.

However, the cultivation of microbes using SSF depends on several technological issues such as oxygen supply for aerobic metabolism; the removal of heat, CO_2_, and volatile components produced from metabolic processes; and the maintenance of suitable moisture content for optimal growth [212,213].

SSF is widely applied in the cultivation of filamentous fungi. The attention of researchers in East and Southeast Asia studying the technological and dietological experience of obtaining traditional food products through the fermentation of soybeans using *Aspergillus* and *Rhizopus* mushrooms has significantly enriched biotechnology worldwide [214]. It was found that in the process of solid-phase fermentation, not only did the protein concentration and the nutritional value increase, the content of anti-nutritional factors of the substrate decreased [205,222,223,224]. The biosynthesis of active substances is also more efficient [225], including antibiotics [226,227], phytohormones, food pigments, and alkaloids. [228,229].

Bacteria and fungi are the two main types of microorganisms used in Asian fermented foods, with most of them, primarily Japanese natto, being fermented with *Bacillus* spp. During fermentation, *Bacillus subtilis* produces various metabolites including peptones, peptides, amino acids, sugars, organic acids, and the enzyme nattokinase [230] that are capable of modulating human and animal health. There is evidence that SSF with several species of the genus *Aspergillus* such as *A. niger* and *A. oryzae* is inferior in its efficiency in increasing the availability of nutrients in soybean substrate and reducing the pool of anti-nutritional factors as compared to bacterial fermentation using *B. subtilis* [231]. Since the discovery of the health benefits of fermented foods [232], the number of publications devoted to this topic are progressively increasing, and new aspects are opening up [230,233,234,235,236].

Bacterial cultures can also be successfully used for the SSF of plant raw materials by microorganisms adapted for the fermentation of lignocellulosic substrates, or, that is to say, capable of secreting lignocellulose-degrading enzymes. Until now, only a few studies have exploited the SSF method for *Bacillus* probiotic production, and there is a lack of comparative information on the production of *Bacillus* spp. probiotics under submerged and SSF conditions. In particular, Zhao et al. (2008) achieved the highest yield of spores (1 × 10^11^ spores/g) when a mixture of 15 g wheat bran and 5 g rice straw powder was used as a growth substrate for SSF by *B. licheniformis* B36. Supplementation of this medium with an additional carbon source, either glucose or sucrose, increased spore production by 35% and 25%, respectively, while additional nitrogen sources, peptone and yeast extract, increased the spore yield by 16% and 24%, respectively [222]. In our studies, *B. amyloliquefaciens* B-1895 [6,201] and *B. subtilis* KATMIRA1933 [13] showed a capability to utilize various inexpensive lignocellulosic wastes/by-products as growth substrates for high-yield spore production. The summarized data [5] showed that in most media, SSF was a suitable method for bacilli cultivation, favoring a significant increase in the number of spores compared to those produced during the same time using submerged fermentation. SSF of wheat bran followed by mandarin peels provided especially high yields of B. subtilis KATMIRA1933 spores (5.7 and 4.9 × 10^11^ spores/g, respectively), whereas EPR from wheat grains and wheat straw promoted spore formation in the SSF by *B. amyloliquefaciens* B-1895 (3.8 and 3.1 × 10^11^ spores/g, respectively). Interestingly, depending on the cultivation method, both bacteria showed different preferences for growth substrates. For example, wheat straw appeared to be the worst growth substrate for sporulation by *B. amyloliquefaciens* B-1895 in submerged fermentation, but it was a preferable source of nutrients in SSF conditions. In the submerged fermentation, mandarin peels appeared to be the superior growth substrate for B. subtilis KATMIRA1933 spore production, whereas *B. amyloliquefaciens* B-1895 was capable of efficiently sporulating following fermentation of the majority of tested materials. Overall, these findings suggest that both bacilli possess sufficiently potent enzymatic systems to deconstruct plant raw materials and provide all necessary nutrients required for abundant bacterial growth, whereas the chemical composition, particle structure, and adhesive properties of these materials favors biofilm formation and efficient sporulation.

#### 7.2.2. Perspectives on Scaling up Fermentation Processes

Scaling up fermentation is the last step in the development of the production process, and several research groups have demonstrated the technical feasibility of scaled-up production of *Bacillus* spp. spores. Sen and Babu (2005) developed a two-stage strategy for *B. coagulans* RK-02 cultivation and sporulation in a 20 L fermenter. During the first stage, cultivation conditions were created that were favorable for the production of biomass, while for the second stage, in the stationary phase, conditions optimal for sporulation were maintained to obtain a maximum spore yield of 9 × 10^11^ spores/g [236]. Monteiro et al. (2014) cultivated *B. subtilis* in a 2 L bioreactor, using an optimized, chemically defined medium, and during the exponential growth phase, the authors increased the agitation rate from 100 to 1200 rpm to compensate for the oxygen consumption rate. The maximum vegetative cell concentration (1.3 × 10^10^ cells/mL) was obtained at the end of the exponential growth phase. Thereafter, cell lysis was observed, but only 48% of vegetative cells produced heat-resistant spores, with a final concentration of 6.3 × 10^9^ spores/mL [200].

Cultivation of *B. subtilis* KATMIRA1933 was performed in a 7 L fermenter filled with an optimized medium containing mandarin peels as a growth substrate [201]. At a fermenter stirring speed of 300 rpm and aeration rate of 1.0 L/L/min, bacilli multiplication proceeded rapidly, and after 24 h of fermentation, the number of vegetative cells increased from 3 × 10^6^ CFU/mL to 2.4 × 10^10^ CFU/mL, with a spore concentration of 3 × 10^8^ spores/mL. During the second day, the vegetative cells and spore numbers increased to 8.1 × 10^10^/mL and 9.3 × 10^9^ spores/mL, respectively. In subsequent cultivation, the *B. subtilis* KATMIRA1933 cell number increased to 1.04 × 10^11^ CFU/mL after 96 h fermentation, with a maximum yield of 6.5 × 1010 spores/mL.

Undoubtedly, a promising strategy for the mass production of probiotics is the use of fed-batch cultures when the concentration of the limiting substrate (usually, carbon source) can be kept very low, thus avoiding the repressive effects of high concentrations of the substrate. In this case, all other nutrients are present in sufficient quantities so that the growth of the microorganism is controlled solely by the concentration of the carbon source present [194,237]. Thus, a fed-batch cultivation process in a 2 L bioreactor was developed for *B. subtilis* spore production with a high yield. Initially, the culture was grown for 5 h in batch mode in a medium containing 3.5 g glucose/L. Before the complete depletion of glucose in the middle of the exponential growth phase, a nutrient feed was started to extend the exponential growth phase, prevent sporulation, and accumulate a maximum concentration of vegetative cells (3.6 × 10^10^ cells/mL). At the end of the fed-batch phase, glucose was completely depleted from the medium, causing a spike in dissolved oxygen concentrations and indicating the onset of the sporulation process. This fed-batch process of *B. subtilis* cultivation resulted in an increase in spore production, with the highest yield of 7.4 × 10^9^ spores/mL. To obtain a high yield of the probiotic *Bacillus coagulans*, Pandey and Vakil (2016) first achieved a high cell density in batch culture followed by fed-batch fermentation in which glucose was added intermittently in portions. The maximum biomass yield reached was 30 g/L, which corresponded to 3.8 × 10^11^ cells/mL, with a high spore titer of 1.9 × 10^11^/mL and a sporulation efficiency of about 81%. High biomass production was achieved by maintaining the dissolved oxygen (DO) concentration above a critical level (20% DO) to meet the organism’s maximum specific oxygen demand [194].

The upscaling of the SSF process to a pilot level for probiotic production was carried out for the first time by Berikashvili et al. (2018) after optimizing the composition of the culture medium for *B. amyloliquefaciens* B-1895. In these experiments, bacilli cultivation in 1 kg of milled corncobs soaked by an optimized cheese whey-containing medium and placed in polypropylene gas-permeable bags resulted in the accumulation of 1.0 × 10^12^ spores per gram of dry biomass [6]. Recently, the feasibility of the developed medium and SSF strategy was proven for *B. subtilis* KATMIRA1933 probiotic production, when bacilli were cultivated in polypropylene bags or trays filled with 2 kg of wheat bran or milled corn cobs and formed 4.9 × 10^11^ spores/g and 4.3 × 10^11^ spores/g, respectively (unpublished results). These findings show that the SSF of plant raw materials by spore-forming bacteria has great potential for the efficient production of cheap probiotics.

In conclusion, the analysis of literature data shows that only a few *Bacillus* spp. have been extensively studied so far, and current knowledge on their physiology is still too limited to effectively realize their biotechnological potential on an industrial scale. Especially little is known about the physiological peculiarities of bacilli growth and spore production during lignocellulose fermentation, under solid-state conditions in particular. Moreover, information on hydrolytic enzyme production by probiotic bacilli during lignocellulose fermentation is limited, although polysaccharides are typically the main resource for bacterial growth, and cellulases play a decisive role in steadily supplying a carbon and energy source to the bacteria. It is necessary to elucidate the physiological mechanisms that regulate (enhance or suppress) the growth and sporulation of individual bacilli as well as understand the optimal nutrient requirements for both processes. Finally, to develop effective technology for the production of spore-forming bacteria, a reasonable strategy for increasing the production of probiotics is to create conditions at the beginning of cultivation that ensure high cell density as well as conditions that allow sporulation to occur.

## 8. Conclusions

**Figure 1:**
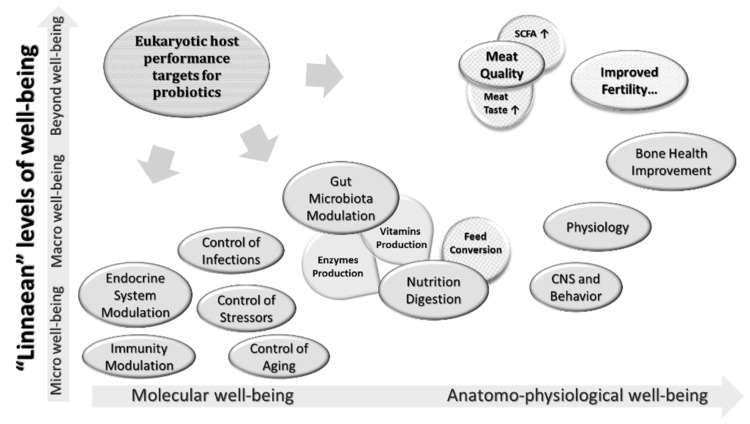
Pseudo-Linnaean representation of spore-forming effects on the poultry industry from the molecular and species levels to full-scale industry.

Spore-forming probiotics have the potential to take a dominant position among novel growth promoters in poultry for their wide range of features from molecular to industrial levels, which are summarized in Figure 1.

However, there are some problems we are facing right now. The first and most important, to our minds, is the lack of reproducibility in poultry probiotics trials, not because of the absence of conscientious study design, randomization, or statistical analysis, but because of the absence of comparability of the evaluated parameters and limited information provided on the probiotic preparations used in these trials. The other major issue is developing effective technology to produce spore-forming bacteria on an industrial level, which now is not achievable due to limited knowledge of their physiology. We should invent ways to create cultivation conditions that ensure high cell density as well as conditions that allow sporulation to occur to achieve the full biotechnological potential of spore-forming probiotics for the poultry industry.

## Data Availability

All data provided in this manuscript were adapted from other published sources and were appropriately cited in the tables, figures, and reference section.

## Appendix A

**Table A1:** Comparison table of studies of spore-forming probiotics effects on performance parameters in poultry.

Animal	Spore-Forming Probiotic Strain, Supplemental Level	Way of Probiotic Administration	Performance Parameters	Results of the Experimental Group Compared to the Control Group	References
Eggs of broiler Cobb 500 (*Gallus gallus domesticus*)	*B. subtilis* fermentation extract (each egg received 1 × 10^7^ CFU of the bacterium/200 mL saline diluent)	In ovo	Hatch performance (pipped eggs, late dead eggs, hatchability, average chick weight, chick body weight/initial egg weight, %)	In ovo injection of saline and probiotics resulted in a significant decrease of pipped eggs compare to intact control eggs.	Oladokun et al., 2021 [238]
			Average daily feed intake (g/bird)	No significant difference.	
			Average daily gain (g/bird)	No significant difference.	
			Feed conversion ratio	No significant difference.	
Broiler Cobb 500 (*Gallus gallus domesticus*)	In-water probiotic formulation containing 2.5 × 10^8^ CFU of *B. subtilis*/L of drinking water	Supplementing drinking water	Average daily feed intake (g/bird)	No significant difference.	
			Average daily gain (g/bird)	No significant difference.	
			Feed conversion ratio	No significant difference.	
	In-feed probiotic formulation containing 5 × 10^8^ CFU of *B. subtilis*/kg of feed	Supplementing standard diet	Average daily feed intake (g/bird)	No significant difference.	
			Average daily gain (g/bird)	No significant difference.	
			Feed conversion ratio	No significant difference.	
Hy-Line Brown laying hens (*Gallus gallus domesticus*)	*B. subtilis* C-3102 spores at 9.3 × 10^9^ CFU/kg	Supplementing standard diet	Egg production, %	No significant difference.	Wang et al., 2021 [239]
			Egg weight, g	No significant difference.	
			Average daily feed intake, g	No significant difference.	
			Food conversion ratio (feed:egg, g:g)	A significant decrease from 2.15 in the control group to 2.08 in the treatment group.	
Hy-Line Brown laying hens (*Gallus gallus domesticus*)	*B. velezensis* (Group I, 1 × 10^10^ CFU/kg; Group II, 2 × 10^10^ CFU/kg)	Supplementing standard diet	Average egg production rate, %	A significant increase from 78.889 ± 0.007 in the control group to 80.827 ± 0.005 (Group I) and 81.905 ± 0.006 (Group II).	Ye et al., 2020 [240]
			Average egg weight, g	A significant increase from 61.972 ± 0.150 in the control group to 60.362 ± 0.140 (Group I) and 61.192 ± 0.111 (Group II).	
			Average daily feed intake, g	A significant increase from 110.608 ± 0.368 in the control group to 112.546 ± 0.281 (Group I) and 111.435 ± 0.229 (Group II).	
			Feed conversion ratio	No significant difference.	
Hy-Line Brown laying hens (*Gallus gallus domesticus*)	*B. amyloliquefaciens* BLCC1-0238 (2 × 1010 CFU/g) at 0.01%, 0.03%, or 0.06% levels	Supplementing standard diet	Egg production, %	A significant increase from 93.8 ± 0.3 in the control group to 97.9 ± 0.5 (0.01%) and 97.7 ± 0.2 (0.06%) in groups treated with spore-forming probiotics.	Zhou et al., 2020 [241]
			Egg weight, g	No significant difference.	
			Egg mass, g/hen per day	Significant increase from 54.0 ± 0.5 in control group to 57.1 ± 0.7 (0.01%) and 57.0 ± 0.4 (0.06%) in groups treated with spore-forming probiotics.	
			Feed intake, g/hen per day	No significant difference.	
			Feed conversion, g/g	No significant difference.	
Lohmann pink hens (*Gallus gallus domesticus*)	*B. subtilis* C-3102 at 5 × 10^8^ CFU/kg (and mixed with montmorillonite, which was not covered by this review)	Supplementing basal diet	Egg production, %	A significant increase from 94.33 in the control group to 95.94 in the group treated with spore-forming probiotics.	Chen et al., 2020 [242]
			Egg mass, g/hen per d	No significant difference.	
			Feed conversion ratio, g of feed/g of egg	No significant difference.	
Shaver White laying hens (*Gallus gallus domesticus*)	*B. subtilis* DSM29784, 1.1 × 10^8^ CFU/kg (low); *B. subtilis* DSM29784, 2.2 × 10^8^ CFU/kg (medium)*, B. subtilis* DSM29784; 1.1 × 10^9^ CFU/kg (high)	Supplementing corn–soybean meal	Body weight (kg)	Significant improvement from 1549 to 1568 (low), 1601 (medium), 1581 (high) observed in week 32. No significant difference in other dates/periods.	Neijat et al., 2019 [10]
			Feed intake (g/day/bird)	Significant improvement from 81.3 to 86.3 (low), 83.5 (high) observed in week 20. No significant difference in other dates/periods.	
			Egg production (% hen-day)	Significant improvement from 31.8 to 38.2 (low), 41.6 (medium) observed in week 19. No significant difference in other dates/periods.	
			Egg weight (g/egg)	A significant decrease from 44.1 for medium dose compared to other doses (44.5 for control, 45.6 for low, 44.6 for high) observed in week 20. No significant difference in other dates/periods.	
			Egg mass (g/egg)	Significant improvement in week 20, resulting in an overall improvement across the layer I phase (tabular values are not presented). No significant difference in the layer II phase.	
			Feed conversion ratio (feed intake, g:egg mass, g)	Significant improvement from 1.59 to 1.62 (high) was observed in week 40. No significant difference in other dates/periods.	
Hisex Brown hens (*Gallus gallus domesticus*)	*B. subtilis* KATMIRA1933 (Group I; 10^7^–10^9^ CFU viable spores per gram of the probiotic supplement), *B. amyloliquefaciens* B-1895 (Group II; 10^7^–10^9^ CFU viable spores per gram of the probiotic supplement), both strains (Group III; equal amounts, 10^7^–10^9^ CFU viable spores per gram of the probiotic supplement)	Supplementing the standard diet via solid phase fermentation	Egg laying, %	Egg laying in the experimental groups exceeded the control by 1.8% (*p* < 0.05) in Group I, 0.62% (*p* < 0.05) in Group II, and 0.36% (*p* < 0.05) in Group III.	Prazdnova et al., 2019 [243]
			Egg weight, g	The weight of eggs in the experimental groups exceeded the control by 3.00% in Group I (*p* < 0.05), 1.99% in Group II (*p* < 0.05), and 2.38% in Group III (*p* < 0.05).	
			Yolk mass, g	The weight of yolk in the experimental groups exceeded the control by 3.49% in Group I (*p* < 0.05), 1.96% in Group II, and 2.28% in Group III.	
			Albumen mass/yolk weight	In the experimental groups, the ratios decreased to 1.90 (*p* < 0.05), 1.92 (*p* < 0.05), and 1.91 (*p* < 0.05) in groups I, II, III, respectively, compared to 1.93 (*p* < 0.05) in the control.	
			Protein index	In the experimental groups, it was significantly higher than the control at 8.77 (*p* < 0.01) in Group I, 6.14 (*p* < 0.05) in Group II, and 7.89 (*p* < 0.01) in Group III.	
			Egg hatching, %	The hatching rate in Groups I and II was 86.76% (*p* < 0.05), which was 2.94% (*p* < 0.05) higher than the control. The hatching rate of Group III was 84.55% (*p* < 0.05), which exceeded the control by 0.73% (*p* < 0.05).	
			Egg fertilization, %	High rates of chickens’ output in experimental Groups I and II were obtained due to the increase in egg fertilization up to 97.06% (*p* < 0.05), and the hatchability of eggs reached a maximum value of 89.39% (*p* < 0.05).	
Hy-LineBrown pullets (*Gallus gallus domesticus*)	GalliProMax/*B. subtilis*, 500 g/ton (GPM) 8 × 10^5^ CFU/g; GalliPro Tect/*B. licheniformis*, 500 g/ton (GMT) 8 × 10^5^ CFU/g	Supplementing the standard diet	Body weight, g	No significant difference.	Upadhaya et al., 2019 [244]
			Body weight gain, g	No significant difference.	
			Feed intake, g	A significant decrease from 3241 in the control group to 3123 (GPM) and an increase to 3312 (GMT).	
			Feed conversion ratio	No significant difference.	
			Egg production, %	A significant increase from 92.07 in the control group to 94.91 (GPM).	
Lohmann pink laying hens (*Gallus gallus domesticus*)	*Clostridium butyricum* (the dose of the dietary probiotic supplement was added with reference to the company’s commercial recommendations)	Supplementing a standard diet (0.5 g/kg of probiotic)	Average daily feed intake	A significant decrease from 105.5 ± 1.80 in the control group to 104.1 ± 1.14 in the treatment group.	Xiang et al., 2019 [245]
			Average egg weight, g	No significant difference.	
			Feed conversion (g of feed/g of egg)	A significant decrease from 1.97 ± 0.04 in the control group to 1.92 ± 0.03 in the treatment group.	
			Laying rate, %	No significant difference.	
			Mortality. %	No significant difference.	
			Average cracked eggs, %	No significant difference.	
Hy-Line Brown laying hens (*Gallus gallus domesticus*)	*B. licheniformis* yb-214245 (BL; 1.0 × 10^6^ CFU/kg), *B. subtilis* yb-114246 (BS; 1.0 × 10^6^ CFU/kg), both strains (CB; (6.6 × 10^5^:3.3 × 10^5^ BL:BS)	Supplementing the standard diet via blending with 99.01 kg basic mass feed.	Laying rate, %	A significant increase from 75.9 in the control group to 79.1 (BL), 82.3 (BS), and 82.2 (CB) in the 60–72 weeks period.	Yang et al., 2019 [246]
			Egg weight, g	No significant difference.	
			Egg mass, g/hen per day	A significant increase from 46.9 in the control group to 49.5 (BL), 50.8 (BS), and 51.0 (CB) in the 60–72 weeks period.	
			Feed consumption, g/hen per day	No significant difference.	
			Feed conversion	A significant decrease from 2.49 in the control group to 2.36 (BL), 2.3 (BS), and 2.28 (CB) in the 60–72 weeks period.	
			Soft broken egg rate, %	A significant decrease from 1.61 in the control group to 0.43 (BL), 0.65 (BS), and 0.33 (CB) in the 60–72 weeks period.	
			Malformed egg rate, %	A significant decrease from 0.17 in control group to 0.12 (BL), 0.10 (BS), and 0.09 (CB) in the 60–72 weeks period.	
Arbor Acres broilerchickens (*Gallus gallus domesticus*)	*B. subtilis* CGMCCN 0943 at 1.0 × 10^11^ CFU/g	Supplementing standard diet at 0.2 g/kg of probiotic (BS-1), at 0.3 g/kg of probiotic (BS-2), at 0.4 g/kg of probiotic (BS-3), at 0.5 g/kg of probiotic (BS-4)	Body weight, g/bird	No significant difference.	Bai et al., 2018 [247]
			Feed intake, g/bird	No significant difference.	
			Feed conversion ratio, g/g	No significant difference.	
ISA brown laying hens (*Gallus gallus domesticus*)	Complex strain of spray-dried spores forming *B. amyloliquefaciens*	Supplementing a standard diet of 1.0 × 10^7^ CFU/kg probiotic (P1) and 2.0 × 10^7^ CFU/kg probiotic (P2)	Egg production, %	Significant improvement from 89.4 in the control group to 91.8 (P1) and 92.0 (P2) at 4–6 weeks.	Tang et al., 2018 [248]
			Egg weight, g	No significant difference.	
Jinghong-1 strain laying hens (*Gallus gallus domesticus*)	*C. butyricum* at 2.5 × 10^4^ (CB1), 5 × 10^4^ (CB2), 1 × 10^5^ (CB3), and 2 × 10^5^ (CB4) CFU/g	Supplementing standard corn–soybean basal diet	Egg production, %	A significant increase from 85.4 in the control group to 91.4 (CB2).	Zhan et al., 2018 [249]
			Egg weight, g/hen per day	No significant difference.	
			Egg mass, g/hen per day	A significant increase from 52.5 in control group to 57.1 (CB2).	
			Feed intake, g/hen per day	No significant difference.	
			Feed conversion ratio	No significant difference.	
Japanese quails (*Coturnix coturnix japonica*)	*B. subtilis* at 10^9^ CFU/g	Supplementing the basal diet	Feed intake, g/bird/day	A significant decrease from 25.924 in the control group to 24.694 in the group treated with spore-forming probiotics in the 9 to 23 week period. A significant decrease from 25.552 in the control group to 23.922 in the group treated with spore-forming probiotics in the 24 to 39 weeks period.	Lemos et al., 2018 [250]
			Egg production, %	A significant increase from 90.102 in the control group to 95.981 in the group treated by spore-forming probiotics in the 9 to 23 weeks period. A significant increase from 89.223 in the control group to 92.961 in the group treated with spore-forming probiotics.	
			Egg weight average, g	A significant increase from 11.03 in the control group to 11.26 in the group treated by spore-forming probiotics in the 9 to 23 weeks period. A significant increase from 12.08 in the control group to 12.82 in the group treated with spore-forming probiotics.	
			Egg mass, g	A significant increase from 9.938 in the control group to 10.807 in the group treated with spore-forming probiotics in the 9 to 23 weeks period. A significant increase from 10.778 in the control group to 11.918 in the group treated with spore-forming probiotics.	
			Feed conversion per egg mass, kg/kg	A significant decrease from 2.292 in the control group to 2.210 in the group treated with spore-forming probiotics in the 9 to 23 weeks period. A significant decrease from 2.362 in the control group to 2.246 in the group treated with spore-forming probiotics.	
			Feed conversion per dozen eggs, kg/dozen	A significant decrease from 0.251 in the control group to 0.228 in the group treated with spore-forming probiotics in the 9 to 23 weeks period. A significant decrease from 0.268 in the control group to 0.238 in the group treated with spore-forming probiotics.	
			Viability of the birds, %	No significant difference.	
Xuefeng black-bone chicken (*Gallus gallus domesticus*)	*B. subtilis* C-3102 at 3.0 × 10^5^ (BS-1), 6.0 × 10^5^ cfu/g (BS-2), and 9.0 × 10^5^ (BS-3) CFU/g.	Supplementing the basal diet	Egg weight, g	A significant increase from 45.00 in the control group to 46.71 (BS-2) and 47.03 (BS-3).	Liu et al., 2019 [251]
			Egg production, %	No significant difference.	
			Feed conversion ratio, g of feed/g of egg	No significant difference.	
			Egg mass, g/day per hen	No significant difference.	
			Fertility, %	A significant increase from 91.59 in the control group to 96.68 (BS-1) and 97.64 (BS-3).	
			Hatchability, %	No significant difference.	
			Hatchability of fertile eggs, %	No significant difference.	
Fertile Cobb chicken (*Gallus gallus domesticus*)	*B. subtilis* at 10^7^ CFU/0.5 mL	In ovo	Average feed intake g/day	No significant difference.	Majidi-Mosleh et al., 2017 [252]
			Average weight gain, g/day	No significant difference.	
			Feed conversion ratio	No significant difference.	
Hi-Sex Brown cross laying hens (*Gallus gallus domesticus*)	*B. subtilis* KATMIRA1933 (10^7^–10^9^ CFU viable spores per gram of the probiotic supplement; Group I), *B. amyloliquefaciens* B-1895 (10^7^–10^9^ CFU viable spores per gram of the probiotic supplement; Group II),and *B. subtilis* KATMIRA1933 and *B. amyloliquefaciens* B-1895 (equal amounts, 10^7^–10^9^ CFU viable spores per gram of the probiotic supplement; Group III)	Supplementing the standard diet via solid phase fermentation	The number of eggs, pcs.	A significant increase from 7419 in the control group to 7538 (Group I), 7469 (Group II), and 7482 (Group III).	Mazanko et al., 2017 [184]
			Egg weight, g	A significant increase from 61.64 ± 0.42 in the control group to 63.49 ± 0.67 (Group I) and 63.11 ± 0.37 (Group III).	
Hisex Brown laying hens (*Gallus gallus domesticus*)	*B. subtilis* at 1.5 × 10^8^ CFU/g of the dried product (and various mixes with distillers, or dried grains with solubles, which were not covered by this review)	Supplementing the standard diet	Feed consumption, g/hen per day	No significant difference.	Abd El-Hack et al., 2016 [253]
			Egg weight, g	No significant difference.	
			Hen-day egg production, %	No significant difference.	
			Feed conversion, g of feed/g of egg	No significant difference.	
			Egg mass, g	A significant increase from 63.65 in the control group to 67.12 in the group treated with spore-forming probiotics.	
			Viability rate, %	No significant difference.	
Hy-Line Brown laying hens (*Gallus gallus domesticus*)	*B. subtilis* CGMCC 1.921 at 1.0 × 10^5^ CFU/g (B1), 1.0 × 10^6^ CFU/g (B2), 1.0 × 10^7^ CFU/g (B3), and 1.0 × 10^8^ CFU/g (B4)	Supplementing the basal diet	Egg production, %	A significant increase from 90.6 in the control group to 94.0 (B1), 94.2 (B3), 94.9 (B4) in the 5 to 8 weeks period. A significant increase from 89.6 in the control group to 93.6 (B2), 93.5 (B3), 93.7 (B4) in the 9 to 12 weeks period. No significant difference in other dates/periods.	Guo et al., 2017 [254]
			Feed intake, g/bird/day	No significant difference.	
			Egg weight, g	No significant difference.	
			Feed:egg ratio, g/g	A significant decrease from 2.04 in the control group to 1.97 (B1), 1.95 (B2), 1.95 (B3), 1.94 (B4) in the 13 to 16 weeks period. A significant decrease from 2.01 in the control group to 1.92 (B1), 1.91 (B2), 1.93 (B3), 1.91 (B4) in the 17 to 20 weeks period. A significant decrease from 2.05 in the control group to 1.93 (B1), 1.96 (B2), 1.96 (B3), 1.94 (B4) in the 21 to 24 weeks period. A significant decrease from 2.04 in the control group to 1.93 (B1), 1.95 (B3), 1.94 (B4) in the 1 to 24 weeks period.	
Japanese quails (*Coturnix Coturnix Japonica*)	*B. subtilis* C-3102 at 0.1% level (minimum dose 1.0 × 10^10^ viable spores per gram)	Supplementing the basal diet	Egg production, hen-day %	A significant increase from 69.09 in the control group to 72.22 in the group treated with spore-forming probiotic.	Manafi et al., 2016 [255]
			Feed conversion ratio, g feed/g egg produced	A significant decrease from 3.57 in the control group to 3.42 in the group treated with spore-forming probiotic.	
			Feed intake, g/quail/day	No significant difference.	
			Egg weight, g	A significant increase from 11.15 in the control group to 11.26 in the group treated by spore-forming probiotic.	
Lohmann Brown laying hens (*Gallus gallus domesticus*)	*B. subtilis* ATCC PTA-6737 at 1 × 10^8^ CFU/kg feed	Supplementing the standard diet	Body weight at 18 days, kg	No significant difference.	Sobczak et al., 2015 [256]
			Body weight at 42 days, kg	Significant improvement from 1.954 ± 0.044 in the control group to 2.004 ± 0.050 (1 × 10^8^ CFU/g of probiotic).	
			Body weight gain, from 18 days to 42 days, kg	Significant improvement from 0.359 ± 0.038 in the control group to 0.405 ± 0.037 (1 × 10^8^ CFU/g of probiotic).	
			Egg weight, g	No significant difference.	
			Egg mass, g/hen	No significant difference.	
			Laying rate, %	No significant difference.	
			Feed intake, g/hen	No significant difference.	
			Feed conversion ratio, g feed/g egg mass	No significant difference.	
Hy-Line layer hybrids (*Gallus gallus domesticus*)	*B. subtilis* PB6 at 0.05% dose	Supplementing corn–soybean cake-based diet	Deposition rate	No significant difference.	Forte et al., 2016 [257]
			Feed efficiency	No significant difference.	
			Egg weight	No significant difference.	
Hy-Line W-36 (*Gallus gallus domesticus*)	*B. subtilis* DSM17299 8 × 10^5^ CFU/g, 4 × 10^5^ CFU/g feed, 3 × 10^5^ CFU/g feed	Delivery in spore form in corn and soybeans	Feed intake, g/hen/day	No significant difference.	Ribeiro Jr. et al., 2014 [258]
			Egg production, g/kg	Significant improvement from 895 in the control group to 918 (8 × 10^5^ CFU/g of probiotics). No significant difference in other groups.	
			Egg weight, g/hen/day	Significant improvement from 59.9 in the control group to 60.8 (8 × 10^5^ CFU/g of probiotics), 60.7 (4 × 10^5^ CFU/g of probiotics), and 60.5 (3 × 10^5^ CFU/g of probiotics).	
			Egg mass, g/hen/day	Significant improvement from 53.7 in the control group to 55.7 (8 × 10^5^ CFU/g of probiotics) and 55.3 (4 × 10^5^ CFU/g of probiotics). No significant difference in other groups.	
			Feed conversion ratio per dozen eggs, kg/dz	No significant difference.	
			Feed conversion ratio per egg mass, g/g	No significant difference.	
			Excreta dry matter content, g/kg	Significant improvement from 59.9 in the control group to 60.8 (8 × 10^5^ CFU/g of probiotics), 60.7 (4 × 10^5^ CFU/g of probiotics) and 60.5 (3 × 10^5^ CFU/g of probiotics).	
Ross 308 broiler chicks (*Gallus gallus domesticus*)	*C. butyricum* (0 or 1 × 10^9^ CFU/kg (500 mg/kg))	Supplementing the standard diet	Average daily feed intake, g/day	A significant increase from 83.7 in the control group to 88.0 in the group treated with a spore-forming probiotic in the period from 1 to 42 days.	Zhao et al., 2013 [259]
			Average daily gain, g/day	A significant increase from 46.7 in the control group to 49.1 in the group treated with a spore-forming probiotic in the period from 1 to 42 days.	
			Feed conversion ratio, g/g	No significant difference.	
			Abdominal fat, %	No significant difference.	
			Intramuscular fat (breast muscle), mg/g	A significant increase from 4.34 in the control group to 7.22 in the group treated with a spore-forming probiotic at 42 days.	
			Intramuscular fat (thigh muscle), mg/g	A significant increase from 4.38 in the control group to 7.20 in the group treated with a spore-forming probiotic at 42 days.	
White laying hens (*Gallus gallus domesticus*)	*B. subtilis* PB6 at 0.5 g/kg and 1.0 g/kg supplement levels	Supplementing the basal diet	Egg production, %	Significant increase in both groups treated with spore-forming probiotics compared to control (no table values provided).	Abdelqader et al., 2013 [183]
			Egg weight, g	Significant increase in both groups treated with spore-forming probiotics compared to control (no table values provided).	
			Egg mass, g/hen	Significant increase in both groups treated with spore-forming probiotics compared to control (no table values provided).	
			Feed intake, g/day	No significant difference.	
			Feed conversion, kg/kg	Significant decrease from 3.0 in control group to 2.8 (0.5 g/kg) and 2.6 (1.0 g/kg) in groups treated with spore-forming probiotic.	
Hy-Line Variety W-36 hens (*Gallus gallus domesticus*)	*B. licheniformis* at 0.01% (2 × 10^6^ CFU/g), 0.02% (4 × 10^6^ CFU/g), 0.03% (6 × 10^6^ CFU/g), 0.06% (1.2 × 10^7^ CFU/g), and 0.09% (1.8 × 10^7^ CFU/g),	Supplementing the basal diet	Egg production, %	Significant increase from 94.0 ± 0.4 in control group to 98.4 ± 0.6 (0.01%) and 97.9 ± 0.2 (0.06%).	Lei et al., 2013 [182]
			Egg weight, g	No significant difference.	
			Egg mass, g/hen per day	Significant increase from 54.3 ± 0.6 in control group to 57.0 ± 0.8 (0.01%) and 57.0 ± 0.5 (0.06%).	
			Feed consumption, g/hen per day	No significant difference.	
			Feed conversion, g/g	No significant difference.	
Lohmann pink laying hens (*Gallus gallus domesticus*)	*B. subtilis* at 9 × 10^9^ CFU/g (and various mixes with *Lactobacillus* bacteria and sodium butyrate, which were not covered by this review)	Supplementing the standard diet	Egg production, %	No significant difference.	Zhang et al., 2012 [260]
			Egg weight, g	No significant difference.	
			Daily egg yield, g/hen per day	No significant difference.	
			Feed consumption, g/hen per day	A significant decrease from 2.13 ± 0.03 in the control group to 2.03 ± 0.02 in the group treated with spore-forming probiotics.	
			Feed conversion ratio, g/g	No significant difference.	
			Damaged egg ratio, %	No significant difference.	
Lingnan Yellow broiler chickens (*Gallus gallus domesticus*)	*C. butyricum* at 2.5 × 10^7^ CFU/kg (CB1), 5 × 10^7^ CFU/kg (CB2), 1 × 10^8^ CFU/kg (CB3).	Supplementing the basal diet	Body weight, g	Significant increase from 387.80 in control group to 444.70 (CB1), 431.40 (CB2), and 427.60 (CB3) at 21 days. Significant increase from 1327.50 in control group to 1414.30 (CB1) and 1402.30 (CB2) at 42 days.	Cao et al., 2012 [261]
			Average daily gain, g	Significant increase from 16.50 in control group to 18.50 (CB1), 19.20 (CB2), and 18.40 (CB3) in the 1–21 days period. Significant increase from 30.60 in control group to 32.40 (CB1) and 32.70 (CB2) in the 1–42 days period.	
			Feed conversion ratio (F:G)	Significant decrease from 2.15 in control group to 2.06 (CB1), 2.06 (CB2), and 2.08 (CB3) in the 1–42 days period.	
Hy-Line Brown laying hens (*Gallus gallus domesticus*).According to the study design, all animals except the control group were kept at heat–stress conditions (34 °C). There was no positive control group treated with probiotics kept at normal room temperature conditions (21 °C).	*B. licheniformis* at 10^6^ CFU/g (H + B_1_) and 10^7^ CFU/g (H + B_2_).	Supplementing the basal diet	Egg production, %	A significant decrease from 79.51 in the control group to 60.07 (H + B_1_) and a significant increase from 50.69 in the group kept at heat–stress conditions to 74.35 (H + B_2_).	Deng et al., 2012 [189]
			Egg weight, g	No significant difference.	
			Feed intake, g/bird per day	A significant decrease from 124.32 in the control group to 102.35 (H + B_1_) and 110.28 (H + B_2_); a significant increase from 95.74 in the group kept at heat–stress conditions to 102.35 (H + B_1_) and 110.28 (H + B_2_).	
Shaoxing ducks (*Anas platyrhynchos domesticus*)	*B. subtilis* at 1 × 10^8^ CFU/kg	Supplementing the basal diet	Live body weight, kg	No significant difference.	Li et al., 2011 [262]
			Egg laying rate, %	A significant increase from 84.767 ± 0.6 in the control group to 88.100 ± 0.9 in the group treated by spore-forming probiotics.	
			Mean egg weight, g	No significant difference.	
			Daily egg mass, g	No significant difference.	
			Feed:egg ratio	No significant difference.	
Hy-Line W-36 strains of white Leghorn laying hens (*Gallus gallus domesticus*)	Mix of B. subtilis CH201 and *B. licheniformis* CH200 at 1000 g/ton and 2000 g/ton	Supplementing the basal diet	Egg production, %	A significant increase from 79.69 in the control group to 84.17 (1000 g ton^−1^).	Aghaii et al., 2010 [263]
			Egg weight, g	No significant difference.	
			Feed consumption, g/hen/day	No significant difference.	
			Egg mass, g/hen/day	A significant increase from 45.90 in the control group to 51.03 (1000 g ton^−1^) and 49.19 (2000 g ton^−1^).	
			Feed conversion ratio, g/g	A significant increase from 2.263 in the control group to 2.032 (1000 g ton^−1^).	
Lohmann Brown layers (*Gallus gallus domesticus*)	Dried *Bacillus subtilis* culture at 9.3 × 10^9^ CFU/kg	Supplementing standard diet of 500 mg/kg, 1000 mg/kg, or 1500 mg/kg of probiotic	Egg production, % eggs/hen per day	No significant difference.	Li et al., 2006 [264]
			Egg weight, g	No significant difference.	
			Egg mass, g/hen per day	A significant increase from 50.58 ± 1.88 in the control group to 51.41 ± 2.51 in the treatment group (500 mg of probiotic/kg) for the 26–42 weeks period. No significant difference in other dates/periods.	
			Feed consumption, g/hen per day	A significant increase from 125.39 ± 5.82 in the control group to 117.42 ± 2.22 (500 mg of probiotic/kg) and 118.62 ± 6.31 (1500 mg of probiotic/kg) for the 43–56 weeks period and from 114.49 ± 2.89 in the control group to 111.14 ± 1.34 (500 mg of probiotic/kg) and 111.52 ± 2.71 (1000 mg of probiotic/kg) for the 26–56 weeks period. No significant difference in other dates/periods.	
			Feed conversion ratio, kg of food/kg of egg	A significant increase from 2.67 ± 0.14 in the control group to 2.41 ± 0.15 (500 mg of probiotic/kg) for the 43–56 weeks period and from 2.35 ± 0.09 in the control group to 2.21 ± 0.07 (500 mg of probiotic/kg) for the 26–56 weeks period. No significant difference in other dates/periods.	
			Damaged egg, %	No significant difference.	
			Mortality, %	No significant difference.	
Hy-Line White laying hens (*Gallus gallus domesticus*)	Mix of B. subtilis CH201 and B. licheniformis CH200 at 1.28 × 10^6^ CFU/g (Group I), 3.2 × 10^6^ CFU/g (Group II), 4.6 × 10^6^ CFU/g (Group III)	Supplementing the standard diet	Egg weight, g	No significant difference.	Mahdavi et al., 2005 [265]
			Egg production, %	No significant difference.	
			Feed consumption, g/hen/day	No significant difference.	
			Feed conversion ratio, g/g	No significant difference.	
			Egg mass, g/hen/day	No significant difference.	

**Table A2:** Comparison table of studies of spore-forming probiotics effects on egg quality characteristics in poultry.

Animal	Spore-Forming Probiotic Strain, Dose	Way of Probiotic Administration	Egg Quality	Results of the Experimental Group Compared to the Control Group	References
Hy-Line Brown laying hens (*Gallus gallus domesticus*)	*B. subtilis* C-3102 spores 9.3 × 10^9^ CFU/kg	Supplementing the standard diet	Eggshell breaking strength, N	A significant increase from 37.64 in the control group to 38.46 in the treatment group at week 79.	Wang et al., 2021 [239]
			Eggshell thickness, mm	A significant increase from 37.64 in the control group to 38.46 in the treatment group at week 79.	
			Shell ratio, %	A significant increase from 9.70 in the control group to 9.78 in the treatment group at week 79.	
			Eggshell weight, g	A significant increase from 6.33 in the control group to 6.43 in the treatment group at week 79.	
			Ca content of eggshell, %	A significant increase from 32.49 in the control group to 35.17 in the treatment group at week 79.	
			*p* content of eggshell, %	No significant difference.	
Female Pharaon quails (*Coturnix japonica*)	*B. subtilis* DSM 32424 at 50 (T1), 75 (T2), and 100 (T3) mg/kg bodyweight (at minimum rate 1 × 10^6^ CFU/g)	Dissolving in drinking water	Yolk acid value, mg of potassium hydroxide per g yolk	No significant difference.	Ermakova et al., 2021 [188]
			Yolk carotenoid content, mcg/g yolk	A significant decrease from 30.08 ± 0.73 in the control group to 27.32 ± 0.77 (T1), 33.68 ± 0.82 (T2), and 22.06 ± 0.38 (T3).	
Hy-Line Brown laying hens (*Gallus gallus domesticus*)	*B. velezensis* (Group I, 1 × 10^10^ CFU/kg; Group II, 2 × 10^10^ CFU/kg)	Supplementing standard diet	Egg weight, g	No significant difference.	Ye et al., 2020 [240]
			Egg shape index	No significant difference.	
			Eggshell color	No significant difference.	
			Eggshell strength (× 10^5^ Pa)	A significant decrease from 4.227 ± 0.086 in the control group to 3.832 ± 0.117 (Group I) and 3.942 ± 0.103 (Group II) in the first phase (day 2–21). No significant difference in other dates/periods.	
			Yolk weight, g	No significant difference.	
			Eggshell weight, g	No significant difference.	
			Yolk color	A significant increase from 6.834 ± 0.190 in the control group to 7.403 ± 0.099 (Group II) in the second phase (22–42 days). No significant difference in other dates/periods.	
			Albumen height, mm	A significant increase from 6.933 ± 0.021 in the control group to 8.116 ± 0.073 (Group I) and 7.521 ± 0.178 (Group II) in the second phase (22–42 days). No significant difference in other dates/periods.	
			Haugh units	A significant increase from 80.464 ± 1.378 in the control group to 89.454 ± 0.415 (Group I) and 85.036 ± 1.606 (Group II) in the second phase (22–42 days). No significant difference in other dates/periods.	
			Triglyceride, mg/g	No significant difference.	
			Cholesterol, mg/g	No significant difference.	
Hy-Line Brown laying hens (*Gallus gallus domesticus*)	*B. amyloliquefaciens* BLCC1-0238 (2 × 10^10^ CFU/g) at 0.01%, 0.03%, or 0.06% levels	Supplementing the standard diet	Albumen height, mm	A significant increase from 6.52 ± 0.12 in the control group to 6.96 ± 0.13 (0.03%) and 6.95 ± 0.12 (0.06%) in groups treated with spore-forming probiotics.	Zhou et al., 2020 [241]
			Yolk color	A significant increase from 6.88 ± 0.10 in the control group to 7.19 ± 0.11 (0.03%) in the group treated with spore-forming probiotics.	
			Haugh units	A significant increase from 79.5 ± 1.3 in the control group to 85.6 ± 1.3 (0.06%) in the group treated with spore-forming probiotics.	
			Eggshell thickness, mm	Significant increase from 0.302 ± 0.003 in control group to 0.331 ± 0.003 (0.01%), 0.343 ± 0.004 (0.03%), and 0.328 ± 0.003 (0.06%) in groups treated with spore-forming probiotics.	
			Eggshell strength, *N*	Significant increase from 33.92 ± 0.06 in control group to 38.13 ± 0.07 (0.01%), 38.49 ± 0.08 (0.03%), and 38.50 ± 0.09 (0.06%) in groups treated with spore-forming probiotics.	
Shaver White laying hens (*Gallus gallus domesticus*)	*B. subtilis* DSM29784, 1.1. × 10^8^ CFU/kg (low), *B. subtilis* DSM29784, 2.2 × 10^8^ CFU/kg (medium)*, B. subtilis* DSM29784, 1.1 × 10^9^ CFU/kg (high)	Supplementing corn–soybean meal	Albumen height (mm)	Significant improvement at higher doses of probiotics during the whole layer I and II phases. No significant difference. Age-related impact on decrease.	Neijat et al., 2019 [10]
			Haugh units	Significant improvement at higher doses of probiotics during the whole layer I and II phases.	
			Yolk color	Significant decreases at week 22 (I phase) and week 40 (II phase) with the lowest value (4,8) due to the inclusion of a high dose of probiotics.	
			Egg shell thickness, mm	No significant difference.	
			Shell-breaking strength (kg)	The lowest at week 20 with the high dose of probiotics (4.518 for high dose vs. 4.889 for control). No significant difference in the layer II phase.	
			Egg component weights (egg yolk, egg shell, and albumen, % of total egg weight)	No significant difference.	
			Shell index	No significant difference. Age related impact to decrease.	
			Total microbial count on egg shell (CFU/mL wash suspension)	No significant difference.	
Hisex Brown hens (*Gallus gallus domesticus*)	*B. subtilis* KATMIRA1933 (Group I; 10^7^–10^9^ CFU viable spores per gram of the probiotic supplement), *B. amyloliquefaciens* B-1895 (Group II; 10^7^–10^9^ CFU viable spores per gram of the probiotic supplement), both strains (Group III; equal amounts, 10^7^–10^9^ CFU viable spores per gram of the probiotic supplement)	Supplementing the standard diet via solid phase fermentation	Yolk mass, g	The weight of yolk in the experimental groups exceeded the control by 3.49% in Group I (*p* < 0.05), 1.96% in Group II, and 2.28% in Group III.	Prazdnova et al., 2019 [243]
			Albumen mass/yolk weight	In the experimental groups, the ratio decreased to 1.90 (*p* < 0.05), 1.92 (*p* < 0.05), and 1.91 (*p* < 0.05) in groups I, II, III, respectively, compared to 1.93 (*p* < 0.05) in the control.	
			Protein index	In the experimental groups, it was significantly higher than the control at 8.77 (*p* < 0.01) in Group I, 6.14 (*p* < 0.05) in Group II, and 7.89 (*p* < 0.01) in Group III.	
			Haugh units	It was 1.78% (*p* < 0.01), 1.47% (*p* < 0.05), and 1.64% (*p* < 0.05) above the control in Group I, Group II and Group III, respectively.	
			Egg shell thickness, mm	In the experimental groups, it exceeded the control by 3.35% (*p* < 0.01) in Group I, 1.96% (*p* < 0.05) in Group II, and 2.79% (*p* < 0.05) in Group III.	
			Dry matter in the albumen portion, %	A significant increase (*p* < 0.01) in Group I (12.892) compared to control (12.026).	
			Dry matter in the yolk portion, %	Significant increase in Group I (52.429; *p* < 0.01), Group II (52.104; *p* < 0.01) and Group III (52.104; *p* < 0.05) compared to control (54.412).	
			Protein content in the albumen portion, %	A significant increase (*p* < 0.01) in Group I (11.414) compared to control (10.605).	
			Protein content in the yolk portion, %	Significant increase in Group I (17.325; *p* < 0.01), Group II (17.239; *p* < 0.01), and Group III (17.272; *p* < 0.01) compared to control (15.820).	
			Fat in the albumen portion, %	No significant difference.	
			Fat in the yolk portion, %	No significant difference.	
			Carbohydrates in the albumen portion, %	No significant difference.	
			Carbohydrates in the yolk portion, %	No significant difference.	
			Amino acid content in eggs, g/100 g	Within the physiological norm.	
Hy-Line Brown laying hens (*Gallus gallus domesticus*)	GalliPro Max/*B. subtilis*, 500 g/ton (GPM) 8 × 10^5^ CFU/g; GalliPro Tect/*B. licheniformis*, 500 g/ton (GMT) 8 × 10^5^ CFU/g	Supplementing the standard diet	Egg weight, g	No significant difference.	Upadhaya et al., 2019 [244]
			Albumen height	A significant increase from 8.43 in the control group to 8.73 (GPM).	
			Yolk color	A significant increase from 5.8 in the control group to 6.12 (GPM).	
			Haugh units	No significant difference.	
			Shell color	No significant difference.	
			Eggshell strength, kg/cm^2^	No significant difference.	
			Eggshell thickness, mm^−2^	No significant difference.	
Lohmann pink laying hens (*Gallus gallus domesticus*)	*Clostridium butyricum* (The dose of the dietary probiotic supplement was added with reference to the company’s recommendations)	Supplementing a standard diet (0.5 g/kg of probiotic)	Egg shape index	No significant difference.	Xiang et al., 2019 [245]
			Eggshell strength, kg/cm^2^	A significant decrease from 4.77 ± 0.27 in the control group to 4.41 ± 0.33 in the treatment group.	
			Haugh units	No significant difference.	
			Albumen height, mm	No significant difference.	
			Yolk color	A significant decrease from 7.33 ± 0.26 in the control group to 7.07 ± 0.27 in the treatment group.	
			Eggshell thickness, um	No significant difference.	
			Yolk percentage, %	No significant difference.	
			Yolk CP%/DM	No significant difference.	
			Albumen CP%/DM	A significant increase from 81.06 ± 1.63 in the control group to 82.65 ± 0.91 in the treatment group.	
			Yolk fat%/DM	No significant difference.	
			Cholesterol content of yolk, %	No significant difference.	
Hy-Line Brown laying hens (*Gallus gallus domesticus*)	*B. licheniformis* yb-214245 (BL; 1.0 × 10^6^ CFU/kg), *B. subtilis* yb-114246 (BS; 1.0 × 10^6^ CFU/kg), both strains (CB; 6.6 × 10^5^:3.3 × 10^5^ BL:BS)	Supplementing the standard diet via blending with 99.01-kg basic mass feed.	Eggshell thickness, mm	No significant difference.	Yang et al., 2019 [246]
			Eggshell strength, kg/cm^2^	A significant increase from 3.36 in the control group to 3.97 (BL), 4.02 (BS), and 4.19 (CB) for the 60–64 weeks period; from 3.30 in the control group to 3.61 (BS) and 4.19 (CB) for the 64–68 weeks period, and from 3.58 in the control group to 3.88 (CB) for the 68–72 weeks period.	
			Albumen height, mm	No significant difference.	
			Haugh units	A significant increase from 85.1 in the control group to 89.7 (CB) for the 60–64 weeks period; from 85.1 in the control group to 89.6 (CB) for the 64–68 weeks period, and from 84.8 in the control group to 89.9 (CB) for the 68–72 weeks period.	
			Egg yolk color	No significant difference.	
			Total cholesterol, mg/g	A significant decrease from 22.39 in the control group to 18.24 (BL), 15.24 (BS), and 14.28 (CB) at 72 weeks.	
			Triglycerides, mg/g	A significant decrease from 9.67 in the control group to 8.20 (BL), 6.07 (BS), and 5.97 (CB) at 72 weeks.	
			Very low-density lipoprotein cholesterol, mg/g	A significant decrease from 12.58 in the control group to 10.17 (BL), 7.08 (BS), and 6.68 (CB) at 72 weeks.	
ISA brown laying hens (*Gallus gallus domesticus*)	Complex strain of spray-dried spores forming *B. amyloliquefaciens* 1.0 × 10^7^ CFU/kg (P1) and 2.0 × 107 CFU/kg (P2)	Supplementing the standard diet	Yolk height, mm	No significant difference.	Tang et al., 2018 [248]
			Yolk color	No significant difference.	
			Haugh units	No significant difference.	
			Eggshell strength, kg/cm^2^	Significant improvement from 3.21 in the control group to 3.55 (P1) and 3.61 (P2) at 3 weeks, and from 3.27 in the control group to 3.47 (P1) and 3.79 (P2) at 6 weeks.	
			Eggshell thickness, mm	Significant improvement from 0.381 in the control group to 0.413 (P1) and 0.426 (P2) at 3 weeks, and from 0.387 in the control group to 0.420 (P1) and 0.441 (P2) at 6 weeks.	
Jinghong-1 strain laying hens (*Gallus gallus domesticus*)	*C. butyricum* at 2.5 × 10^4^ (CB1), 5 × 10^4^ (CB2), 1 × 10^5^ (CB3), and 2 × 10^5^ (CB4) CFU/g	Supplementing standard corn–soybean basal diet	Albumen height, mm	No significant difference.	Zhan et al., 2018 [249]
			Haugh units	No significant difference.	
			Yolk color	No significant difference.	
			Eggshell strength, kgf^3^	A significant increase from 2.40 in the control group to 3.55 (CB2).	
			Eggshell thickness, mm	No significant difference.	
Xuefeng black-bone chicken (*Coturnix coturnix japonica*)	*B. subtilis* C-3102 at 3.0 × 10^5^ (BS-1), 6.0 × 10^5^ cfu/g (BS-2), and 9.0 × 10^5^ (BS-3) CFU/g.	Supplementing the basal diet	Eggshell-breaking strength, kgf	No significant difference.	Liu et al., 2019 [251]
			Eggshell thickness, mm	Significant increase from 0.33 in control group to 0.35 (BS-1) and 0.36 (BS-2).	
			Egg-shape index	No significant difference.	
			Yolk index	No significant difference.	
			Yolk percentage, %	No significant difference.	
			Yolk color	A significant increase from 5.61 in the control group to 6.50 (BS-1) and 6.97 (BS-3).	
			Haugh units	No significant difference.	
Hisex Brown cross laying hens (*Gallus gallus domesticus*)	*B. subtilis* KATMIRA1933 (10^7^–10^9^ CFU viable spores per gram of the probiotic supplement; Group I); *B. amyloliquefaciens* B-1895 (10^7^–10^9^ CFU viable spores per gram of the probiotic supplement; Group II) and *B. subtilis* KATMIRA1933 and *B. amyloliquefaciens* B-1895 (equal amounts, 10^7^–10^9^ CFU viable spores per gram of the probiotic supplement; Group III)	Supplementing the standard diet via solid phase fermentation	Weight of egg albumen g	No significant difference.	Mazanko et al., 2017 [184]
			Weight of egg yolk, g	A significant increase from 18.89 ± 0.17 in the control group to 19.55 ± 0.19 (Group I).	
			Weight of eggshell, g	A significant increase from 6.27 ± 0.09 in the control group to 6.79 ± 0.08 (Group I), 6.61 ± 0.07 (Group II), and 6.73 ± 0.08 (Group III).	
			Shape index, %	No significant difference.	
			Albumen index, %	A significant increase from 9.12 ± 0.14 in the control group to 9.92 ± 0.16 (Group I), 9.68 ± 0.11 (Group II), and 9.84 ± 0.15 (Group III).	
			Yolk index, %	A significant increase from 44.85 ± 0.69 in the control group to 48.83 ± 0.54 (Group I), 48.18 ± 0.61 (Group II) and 48.51 ± 0.47 (Group III).	
			Haugh units	Significant increase from 81.47 ± 0.27 in control group to 82.92 ± 0.33 (Group I), 82.67 ± 0.28 (Group II), and 82.81 ± 0.36 (Group III).	
			Shell thickness, μm	A significant increase from 358.00 ± 2.14 in the control group to 370.00 ± 2.28 (Group I), 365.00 ± 2.11 (Group II), and 368.00 ± 1.99 (Group III).	
			The ratio of egg albumen, %	No significant difference.	
			The ratio of egg yolk, %	No significant difference.	
			Ratio of egg shell, %	No significant difference.	
			The ratio of albumen to yolk	A significant increase from 1.93 ± 0.015 in the control group to 1.90 ± 0.018 (Group I).	
Hisex Brown laying hens (*Gallus gallus domesticus*)	*B. subtilis* at 1.5 × 10^8^ CFU/g of the dried product (and various mixes with distillers, or dried grains with solubles, which were not covered by this review)	Supplementing the standard diet	Egg shape index	No significant difference.	Abd El-Hack et al., 2016 [252]
			Yolk index	No significant difference.	
			Egg shell thickness	A significant increase from 0.370 in the control group to 0.385 in the group treated with spore-forming probiotics.	
			Haugh units	No significant difference.	
			Yolk color, lightness (with a greater value indicating a lighter color)	A significant decrease from 62.13 in the control group to 60.60 in the group treated with spore-forming probiotics.	
			Yolk color, redness (with a greater value indicating a redder color)	A significant increase from 8.88 in the control group to 10.53 in the group treated with spore-forming probiotics.	
			Yolk color, yellowness (with a greater value indicating a more yellow color)	A significant increase from 38.85 in the control group to 43.22 in the group treated with spore-forming probiotics.	
Hy-Line Brown laying hens (*Gallus gallus domesticus*)	*B. subtilis* CGMCC 1.921 at 1.0 × 10^5^ CFU/g (B1), 1.0 × 10^6^ CFU/g (B2), 1.0 × 10^7^ CFU/g (B3), and 1.0 × 10^8^ CFU/g (B4).	Supplementing the basal diet	Eggshell strength	A significant increase from 45.66 in the control group to 52.31 (B1), 51.05 (B3) at 8 weeks. A significant increase from 48.45 in the control group to 53.24 (B1) at 16 weeks. A significant increase from 48.18 in the control group to 51.42 (B1), 51.24 (B2), and 51.79 (B3) at 16 weeks. A significant increase from 48.18 in the control group to 51.42 (B1), 51.24 (B2), and 51.79 (B3) at 20 weeks. A significant increase from 49.40 in the control group to 54.84 (B1) at 24 weeks. No significant difference in other dates/periods.	Guo et al., 2017 [254]
			Albumen height	A significant decrease from 7.8 in the control group to 6.9 (B3) and 7.0 (B4) at 4 weeks. A significant increase from 8.1 in the control group to 8.3 (B1) at 20 weeks. No significant difference in other dates/periods.	
			Yolk color	A significant decrease from 7.8 in the control group to 6.8 (B4) at 0 weeks. A significant increase from 6.7 in the control group to 7.6 (B2), 7.4 (B3), 7.4 (B4) at 1 week. A significant increase from 6.4 in the control group to 7.2 (B2) at 2 weeks. A significant increase from 6.7 in the control group to 6.1 (B2) at 4 weeks. A significant increase from 6.7 in the control group to 7.2 (B2) at 8 weeks. A significant increase from 6.2 in the control group to 6.7 (B3) and 6.5 (B4) at 12 weeks. A significant decrease from 7.1 to 6.7 (B1) and 6.7 (B3) at 16 weeks. A significant increase from 6.3 in the control group to 6.7 (B2) and 6.6 (B4) at 24 weeks. No significant difference in other dates/periods.	
			Haugh units	A significant increase from 88.1 in the control group to 82.6 (B3) and 83.6 (B4) at 4 weeks. No significant difference in other dates/periods.	
Japanese quails (*Coturnix Coturnix Japonica*)	*B. subtilis* C-3102 at 0.1% level (minimum dose 1.0 × 10^10^ viable spores per gram)	Supplementing the basal diet	Eggshell thickness, mm	No significant difference.	Manafi et al., 2016 [255]
			Eggshell-breaking strength, kg	No significant difference.	
			Haugh units	No significant difference.	
			Eggshell, %	No significant difference.	
Lohmann Brown laying hens (*Gallus gallus domesticus*)	*B. subtilis* ATCC PTA- 6737 at 1 × 10^8^ CFU/kg feed	Supplementing the standard diet	Shell thickness, mm	Significant improvement from 0.355 ± 0.008 in the control group to 0.365 ± 0.008 (1 × 10^8^ CFU/g of probiotic).	Sobczak et al., 2015 [256]
			Shell strength, N	Significant improvement from 45.12 ± 2.30 in the control group to 47.63 ± 2.78 (1 × 10^8^ CFU/g of probiotic).	
			Yolk color, points	Significant improvement from 7.83 ± 0.83 in the control group to 9.01 ± 0.71 (1 × 10^8^ CFU/g of probiotic).	
			Haugh units	Significant improvement from 70.45 ± 3.45 in the control group to 72.95 ± 2.59 (1 × 10^8^ CFU/g of probiotic).	
			Egg composition–yolk, %	No significant difference.	
			Egg composition–albumen, %	No significant difference.	
			Egg composition–shell, %	A significant increase from 9.79 ± 0.18 in the control group to 10.04 ± 0.15 (1 × 10^8^ CFU/g of probiotic).	
			Fatty acid profile of egg yolk, (% of total fatty acid content)	Significant increase of oleic acid content from 1.78 ± 0.12 in the control group to 1.93 ± 0.15 (1 × 10^8^ CFU/g of probiotic). No significant difference in other fatty acids.	
			Content in egg yolk fat (cholesterol), mg/g	A significant decrease from 28.1 ± 2.0 in the control group to 24.8 ± 4.6 (1 × 10^8^ CFU/g of probiotic).	
Hy-Line layer hybrids (*Gallus gallus domesticus*)	*B. subtilis* PB6 at 0.05% dose	Supplementing corn–soybean cake-based diet	Yolk weight, g	No significant difference. No significant difference.	Forte et al., 2016 [257]
			Albumen weight, g	No significant difference.	
			Shell weight, g	No significant difference.	
			Shell ash, %	No significant difference.	
			Yolk, %	No significant difference.	
			Albumen, %	No significant difference.	
			Shell, %	No significant difference.	
			Edible, %	No significant difference.	
			Albumen/yolk	No significant difference.	
			Color (Roche scale)	No significant difference.	
			Haugh units	No significant difference.	
			Color, lightness	No significant difference.	
			Color, redness	No significant difference.	
			Color, yellowness	No significant difference.	
			Ash, %	No significant difference.	
			Crude protein, %	No significant difference.	
			Lipid, %	No significant difference.	
			Cholesterol, mg/g yolk	No significant difference.	
			Cholesterol, mg/egg	No significant difference.	
Hy-Line W-36 (*Gallus gallus domesticus*)	*B. subtilis* GalliPro^®^ patented by Chr. Hansen 8 × 10^5^ CFU/g (T2), 4 × 10^5^ CFU/g feed (T3), 3 × 10^5^ CFU/g feed (T4)	Delivery in spore form in corn and soybeans	Yolk weight, g/kg	No significant difference.	Ribeiro Jr. et al., 2014 [258]
			Eggshell weight, g/kg	No significant difference.	
			Albumen weight, g/kg	No significant difference.	
White laying hens (*Gallus gallus domesticus*)	*B. subtilis* PB6 at 0.5 g/kg and 1.0 g/kg supplement levels	Supplementing the basal diet	Eggshell weight, % of egg weight	Significant increase in both groups treated with spore-forming probiotics compared to control (no table values provided).	Abdelqader et al., 2013 [183]
			Eggshell thickness, mm	Significant increase in both groups treated with spore-forming probiotics compared to control (no table values provided).	
			Eggshell density, mg/cm^2^	Significant increase in both groups treated with spore-forming probiotics compared to control (no table values provided).	
			Unmarketable eggs, %	Significant decrease in both groups treated with spore-forming probiotics compared to control (no table values provided).	
Hy-Line Variety W-36 hens (*Gallus gallus domesticus*)	*B. licheniformis* at 0.01% (2 × 10^6^ CFU/g), 0.02% (4 × 10^6^ CFU/g), 0.03% (6 × 10^6^ CFU/g), 0.06% (1.2 × 10^7^ CFU/g), and 0.09% (1.8 × 10^7^ CFU/g),	Supplementing the basal diet	Albumen height, mm	Significant increase from 6.50 ± 0.15 in control group to 6.95 ± 0.12 (0.03%) and 6.93 ± 0.13 (0.06%).	Lei et al., 2013 [182]
			Yolk color	Significant increase from 6.50 ± 0.15 in control group to 6.95 ± 0.12 (0.03%) and 6.93 ± 0.13 (0.06%).	
			Haugh units	Significant increase from 6.86 ± 0.09 in control group to 7.20 ± 0.10 (0.03%) and 6.51 ± 0.0 (0.09%).	
			Eggshell thickness, mm	Significant increase from 0.303 ± 0.004 in control group to 0.332 ± 0.004 (0.01%), 0.324 ± 0.003 (0.02%), 0.342 ± 0.005 (0.03%), 0.327 ± 0.004 (0.06%), and 0.319 ± 0.003 (0.09%).	
			Eggshell strength, N	Significant increase from 33.91 ± 0.08 in control group to 38.12 ± 0.08 (0.01%), 37.44 ± 0.08 (0.02%), 38.51 ± 0.09 (0.03%), 38.51 ± 0.10 (0.06%), and 36.85 ± 0.06 (0.09%).	
Lohmann pink layer hens (*Gallus gallus domesticus*)	*B. subtilis* at 9 × 10^9^ CFU/g (and various mixes with *Lactobacillus* bacteria and sodium butyrate, which were not covered by this review)	Supplementing the standard diet	Yolk color	No significant difference.	Zhang et al., 2012 [261]
			Yolk relative weight, %	A significant decrease from 28.38 ± 0.50 in the control group to 26.49 ± 0.64 in the group treated with spore-forming probiotics.	
			Yolk cholesterol, mg/g yolk	No significant difference.	
			Haugh unit	No significant difference.	
			Shape index	No significant difference.	
			Shell thickness, mm	No significant difference.	
Shaoxing ducks (*Anas platyrhynchos domesticus*)	*B. subtilis* at 1 × 10^8^ CFU/kg	Supplementing the basal diet	Egg weight, kg	No significant difference.	Li et al., 2011 [262]
			Shell thickness, mm	No significant difference.	
			Horizontal–vertical	No significant difference.	
			Egg yolk color	No significant difference.	
			Haugh units	No significant difference.	
			Triglyceride, mmol/L	A significant decrease from 712.45 ± 22.12 to 622.66 ± 28.95 in the group treated with spore-forming probiotics.	
			Total cholesterol, mmol/L	A significant decrease from 126.96 ± 2.79 to 97.09 ± 2.29 in the group treated with spore-forming probiotics.	
			Malondialdehyde	A significant decrease from 943.92 ± 38.68 to 564.99 ± 39.99 in the group treated with spore-forming probiotics.	
Hy-Line W-36 strains of white Leghorn laying hens (*Gallus gallus domesticus*)	Mix of *B. subtilis* CH201 and *B. licheniformis* CH200 at 1000 g/ton and 2000 g/ton	Supplementing the basal diet	Shell thickness, mm	No significant difference.	Aghaii et al., 2010 [263]
			Shell hardness, kg cm^−1^	No significant difference.	
			Haugh units	No significant difference.	
			Yolk index	A significant increase from 0.402 in the control group to 0.420 (1000 g ton^−1^).	
Lohmann Brown layers (*Gallus gallus domesticus*)	Dried *B. subtilis* culture at 9.3 × 10^9^ CFU/kg	Supplementing standard diet with 500 mg/kg, 1000 mg/kg, or 1500 mg/kg of probiotics	Shell strength, kg/cm^2^	No significant difference.	Li et al., 2006 [264]
			Shell thickness, um	No significant difference.	
			Yolk color	No significant difference.	
			Haugh units	No significant difference.	
			Yolk cholesterol (mg/yolk)	A significant decrease from 251.80 ± 13.11 in the control group to 221.05 ± 16.23 in the treatment group (500 mg of probiotic/kg).	
Hy-Line White laying hens (*Gallus gallus domesticus*)	Mix of *B. subtilis* CH201 and *B. licheniformis* CH200 at 1.28 × 10^6^ CFU/g (Group I), 3.2 × 10^6^ CFU/g (Group II), 4.6 × 10^6^ CFU/g (Group III)	Supplementing the standard diet	Shell thickness, mm	No significant difference.	Mahdavi et al., 2005 [265]
			Shell hardness, kg cm^−1^	No significant difference.	
			Haugh unit	No significant difference.	
			Egg cholesterol, mg gr^−1^ yolk	Significant decrease from10.73 in the control group to 10.27 in Group II and 0.23 in Group III.	

**Table A3:** Comparison table of studies of spore-forming probiotics effects on sperm quality characteristics in poultry.

Animal	Spore-Forming Probiotic Strain, Dose	Way of Probiotic Administration	Sperm Quality	Results of the Experimental Group Compared to Control Group	References
Hisex Brown hens (*Gallus gallus domesticus*)	*B. subtilis* KATMIRA1933 (Group I), *B. amyloliquefaciens* B-1895 (Group II), both strains (Group III)	Supplementing standard diet via solid phase fermentation	Color	Within the physiological norm (white among all groups).	Prazdnova et al., 2019 [243]
			The volume of ejaculate, ml	No significant difference.	
			Total number of spermatozoa in the ejaculate, billions	A significant increase from 1.47 to 1.64 (Group I) observed in cocks of age 82 weeks.	
			Concentration of spermatozoa, billion/ml	A significant increase from 2.49 to 2.89 (Group I) observed in cocks of age 82 weeks.	
			The number of morphologically abnormal germ cells in the ejaculate, %	A significant decrease from 15.40 to 11.50 (Group I), 11.98 (Group II), and 12.01 (Group III) observed in cocks of age 82 weeks.	
			Amino acid content in rooster’s semen, g/100 g	The content of amino acids in the sperm of the experimental groups was higher than in the control. A more significant difference in the amino acid composition of the sperm of roosters with respect to the control was observed in Group I with aspartic acid (17.69%, *p* < 0.05), glutamic acid (9.47%, *p* < 0.05), serine (25.87%, *p* < 0.05), and alanine (31.09%, *p* < 0.05).	
Hisex Brown cross laying hens (*Gallus gallus domesticus*)	*B. subtilis* KATMIRA1933 (10^7^–10^9^ CFU viable spores per gram of the probiotic supplement; Group I); *B. amyloliquefaciens* B-1895 (10^7^–10^9^ CFU viable spores per gram of the probiotic supplement; Group II) and *B. subtilis* KATMIRA1933 and *B. amyloliquefaciens* B-1895 (equal amounts, 10^7^–10^9^ CFU viable spores per gram of the probiotic supplement; Group III)	Supplementing the standard diet via solid phase fermentation	Color	Within the physiological norm (white among all groups).	Mazanko et al., 2017 [184]
			The volume of ejaculate, ml	A significant increase from 18.89 ± 0.17 in the control group to 19.55 ± 0.19 (Group I).	
			Total number of spermatozoa in the ejaculate, 10^9^	A significant increase from 1.49 ± 0.05 in the control group to 1.75 ± 0.06 (Group I).	
			Concentration of spermatozoa, 10^9^/mL	A significant increase from 2.56 ± 0.08 in the control group to 3.29 ± 0.07 (Group I), 3.01 ± 0.09 (Group II), and 3.17 ± 0.09 (Group III).	
			The number of morphologically abnormal germ cells in the ejaculate, %	A significant decrease from 14.7 ± 0.40 in control group to 10.4 ± 0.51 (Group I), 11.7 ± 0.43 (Group II), and 10.1 ± 0.62 (Group III).	
White Leghorn roosters (*Gallus gallus domesticus*)	4.5 × 10^4^ CFU of *B. subtilis*/g of feed	Supplementing the standard diet	Sperm quality index	No significant difference.	dos Santos et al., 2018 [266]
			Dead sperm, %	No significant difference.	
			Sperm concentration (total), billion sperm/mL	No significant difference.	
			Sperm concentration (live), billion sperm/mL	No significant difference.	
			Volume, mL	No significant difference.	
			Ejaculated sperm (total), billion sperm/ejaculate	No significant difference.	
			Ejaculated sperm (live), billion sperm/ejaculate	No significant difference.	
			pH	No significant difference.	
			O_2_, nmol/mL	No significant difference.	
			CO_2_, nmol/mL	No significant difference.	
			Na+, μmol/mL	No significant difference.	
			K+, μmol/mL	No significant difference.	
			Ca^2^+, μmol/mL	No significant difference.	
			Cl−, μmol/mL	No significant difference.	
Cobb male broiler breeders (*Gallus gallus domesticus*)	*Bacillus amyloliquefaciens* TOA5001 at 1 × 10^8^ CFU/g	Probiotic in rice supplemented to the standard diet	Sperm count, million/mL	A significant increase from 22.8 ± 2.55 in the control group to 26.5 ± 2.90 in the group treated with spore-forming probiotics.	Inatomi et al., 2018 [267]
			Live sperm, %	A significant increase from 94.1 ± 2.63 in the control group to 95.2 ± 1.06 in group treated with spore-forming probiotics	
